# Reversible Hydration Tuning of Polar Order and Large Nonlinear Optical Response in a 2D van der Waals Crystal, LiInP_2_S_6_

**DOI:** 10.1002/adma.74177

**Published:** 2026-07-31

**Authors:** Jadupati Nag, Saugata Sarker, Michael J. Waters, Safdar Imam, Himirkanti Sarkar, Anthony Richardella, James M. Rondinelli, Mercouri G. Kanatzidis, Venkatraman Gopalan

**Affiliations:** ^1^ Department of Materials Science and Engineering Pennsylvania State University Millennium Science Complex Building University Park Pennsylvania USA; ^2^ Department of Materials Science and Engineering Northwestern University Evanston Illinois United States; ^3^ Department of Chemistry Northwestern University Evanston Illinois United States

**Keywords:** hidden polar phase, hydration engineering, nonlinear optics, second‐harmonic generation, symmetry breaking, van der Waals material

## Abstract

Typically, moisture degrades the performance of commercial optical crystals that are hygroscopic. In contrast, a hydration‐driven symmetry transformation is discovered in a layered van der Waals compound that induces a new polar phase that exhibits a large nonresonant nonlinear optical response. Water intercalation converts the chiral‐nonpolar phase of pristine ${\mathrm{LiInP}}_{2}S_{6}$ (point group 32 and an indirect gap of ∼3.0 eV) into ${\mathrm{LiInP}}_{2}S_{6}\cdot 3H_{2}O$ that exhibits a chiral‐polar structure (point group 3 and an indirect gap of ∼ 3.15 eV), stabilized by sub‐angstrom lattice distortions. This structural change results in new symmetry, allowing nonresonant optical second‐harmonic generation (SHG) coefficients in the hydrated phase of $d_{33}\approx 47$ pm/V and $d_{15}\approx 35$ pm/V at the 1550 nm telecom wavelength; these are up to twice as large as other well‐known materials with similar bandgaps. Density functional theory calculations predict the emergence of a $\Gamma_{2}^{-}$ polar mode consistent with the trigonal point group 3 in hydrated ${\mathrm{LiInP}}_{2}S_{6}$, as well as inform the emergence of large SHG coefficients. The reversible structural transformation via hydration and dehydration is confirmed through temperature‐dependent SHG, x‐ray diffraction, and differential scanning calorimetry. These findings demonstrate intercalation as a powerful means for tuning polar order, enhancing the nonlinear optical response and on‐demand creation and erasure of tunable optical elements for photonic integrated circuits.

## Graphical abstract

Hydration‐induced symmetry breaking transforms layered ${\mathrm{LiInP}}_{2}S_{6}$ from a nonpolar to a chiral‐polar phase, producing a giant enhancement of non‐resonant second‐harmonic generation. The reversible tuning of polar order through molecular intercalation establishes a strategy for dynamically controlling nonlinear optical functionalities in van der Waals materials.

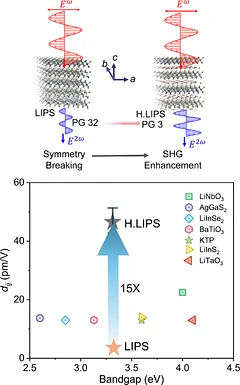

## 1 Introduction

The evolution of emergent crystallographic order driven by compositional tuning [1], chemical intercalation, and strain modulation [2] can induce symmetry transformations leading to enhanced functional properties such as ferroelectricity [3], magnetism [4], superconductivity [5], and nonlinear optical [6] effects. In condensed matter systems, such control parameters can be leveraged to induce polar ground states featuring switchable dipoles and strong nonlinear optical responses [7, 8, 9]. Often, new properties emerge from subtle atomic distortions on the order of a few picometers, which can be challenging to detect and require complementary techniques [10, 11].

This work demonstrates a novel mechanism in a prototype van der Waals (vdW) system, where chemical intercalation drives subtle structural distortions, introducing a polar mode that induces large new symmetry‐allowed nonresonant SHG coefficients, as well as an order of magnitude enhancement of some of the existing SHG coefficients. SHG polarimetry measurements sensitively probe the polar point group (PG) accompanying picometer structural distortions that were challenging to detect with conventional crystallography [12, 13, 14]. Inversion symmetry breaking is sometimes difficult to detect with x‐ray diffraction (XRD), whereas the complementary SHG measurements provide a more local and sensitive probe of broken inversion symmetry in the bulk and can reveal symmetry‐specific responses associated with crystallographic distortions [15], defects [16], and interfaces [17].

Layered thiophosphates possess weak interlayer interactions and a highly compliant bonding framework [18, 19, 20]. Within this family, tetravalent metal thiophosphates of the form $M_{x}P_{2}S_{6}$ ($M_{x}$ = one or more metals) exhibit diverse functionalities [21], including magnetism [22], ferroelectricity [23], ionic conduction [24], and strong light–matter coupling [25]. In Li‐based thiophosphates, the presence of alkali ions and weak interlayer forces makes them highly responsive to environmental stimuli [26, 27, 28]. Recent reports have shown that ${\mathrm{LiInP}}_{2}S_{6}$ can spontaneously absorb water molecules from the ambient air, leading to substantial lattice expansion and drastically enhanced ionic conductivity [26, 27, 28]. Furthermore, intercalation‐driven swelling has been exploited to exfoliate large‐area few‐layer ${\mathrm{LiInP}}_{2}S_{6}$ nanosheets within seconds by a simple manual‐shaking process [26]. These studies reveal how hydration can act as a structural switch, modifying the vdW gap and enabling fast ion transport; however, the implications of hydration for symmetry, polar order, and nonlinear optical functionality remain unexplored. Moreover, while the anhydrous structure of ${\mathrm{LiInP}}_{2}S_{6}$ has been qualitatively described in prior studies, the hydrated phase was previously incorrectly assigned to the centrosymmetric space group $P\bar{3}1c$; [28] its structure remains unresolved thus far.

Here, we show that water intercalation is a direct driver of symmetry lowering, transforming the non‐polar ${\mathrm{LiInP}}_{2}S_{6}$ (PG 32) into a chiral‐polar hydrated phase of ${\mathrm{LiInP}}_{2}S_{6}\cdot 3H_{2}O$ (PG 3). This hydrated phase is stabilized by sub‐Å lattice distortions predicted to be ∼3.1 pm/f.u. by density functional theory (DFT). Temperature‐dependent SHG measurements directly track the reversible point group change between 32 ↔ 3 with hydration (3) and dehydration (32), establishing SHG as a highly sensitive probe of inversion symmetry breaking. This hydration is accompanied by the emergence of new non‐resonant, phase‐matched nonlinear optical coefficients ($d_{33}∼47$ pm/V and $d_{15}∼35$ pm/V) that surpass other well‐known optical crystals with similar electronic bandgaps in the 2.5–4 eV range [25, 29, 30]. These results demonstrate intercalation as a versatile strategy for controlling symmetry, polarity, and nonlinear optical functionality in van der Waals chalcogenides.

## 2 Results and Discussion

${\mathrm{LiInP}}_{2}S_{6}$ is a two‐dimensional vdW material, featuring layers of ordered cations in octahedral coordination situated between bipyramidal P_2_S_6_^4−^ units. In the anhydrous phase, ${\mathrm{LiInP}}_{2}S_{6}$ crystallizes in the trigonal *P*312 space group, with lattice parameters *a* = *b* = 6.09 Å, *c* = 13.02 Å, α = β = 90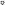, and γ = 120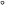. Previous structural refinement work by Qian et al. [27] evaluated alternative space group candidates, including *P*3, *P* $\bar{3}1c$, and *P* $\bar{3}$, but these yielded poor fit statistics, further supporting the assignment of the *P* 312 space group. Although the anhydrous crystal structure of this material has been qualitatively discussed in prior work, the hydrated phase was previously assigned to the centrosymmetric space group $P\bar{3}1c$ [28]. We note that this assignment may arise from symmetry constraints employed during structural modeling, which may not fully capture subtle hydration‐induced symmetry lowering. Our comprehensive XRD, differential scanning calorimetry (DSC), DFT, and SHG studies reveal a previously unrecognized structural phase in ${\mathrm{LiInP}}_{2}S_{6}$, clarifying its coexistence and reversible transformation between polar and nonpolar phases. Finally, phase‐matching calculations are carried out to explore its potential for frequency conversion efficiency.

### 2.1 Hydration‐Induced Reversible Structural Transformation

**FIGURE 1:**
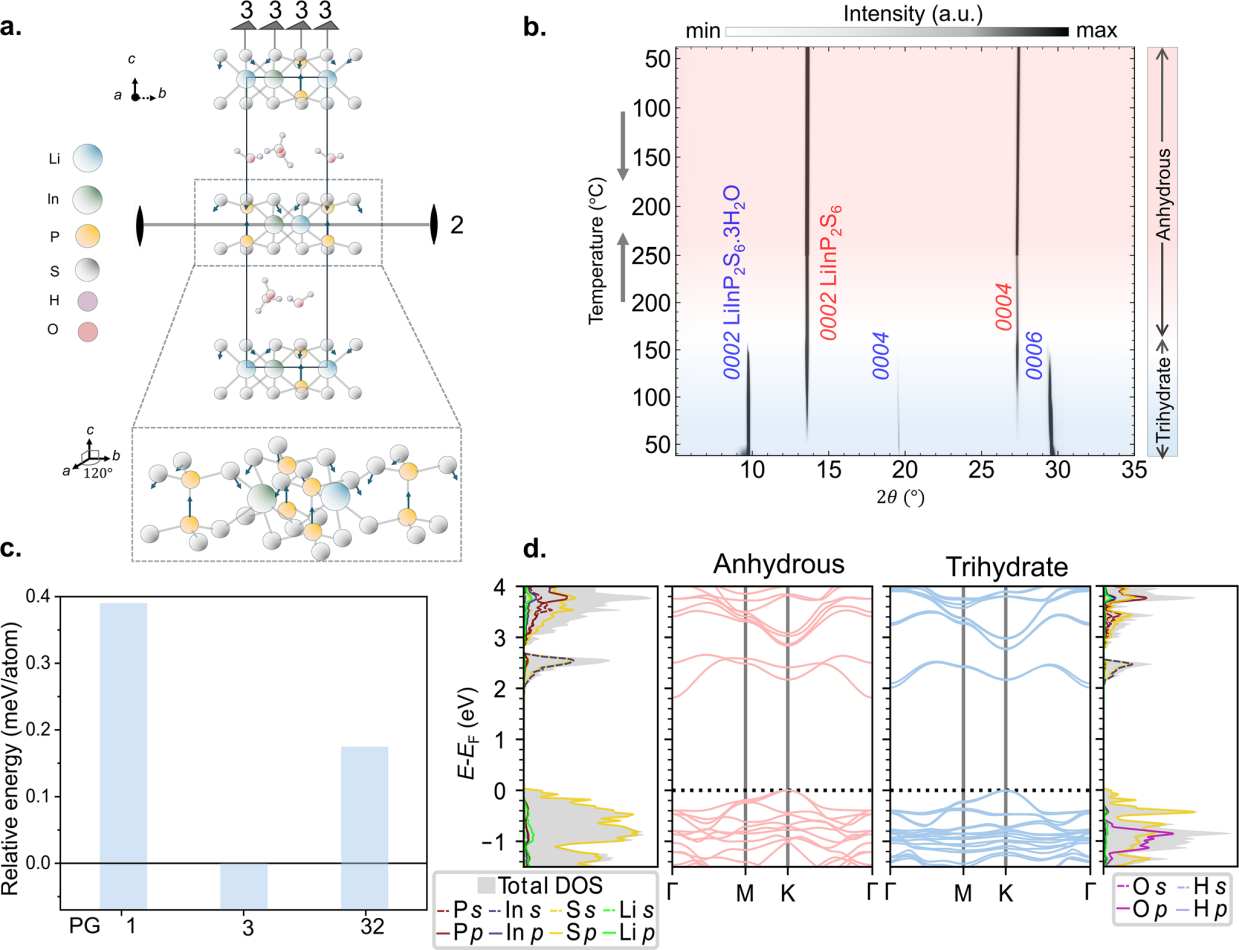
(a) Crystal structure of hydrated ${\mathrm{LiInP}}_{2}S_{6}$ (${\mathrm{LiInP}}_{2}S_{6}\cdot 3H_{2}O$) viewed perpendicular to the *a* axis. Water molecules are intercalated within the interlayer region of ${\mathrm{LiInP}}_{2}S_{6}$. The locations of twofold (labeled 2) and the threefold (labeled 3) rotation axes of the anhydrous phase are indicated; in the hydrated phase. DFT calculations identify $H_{2}O$ intercalation as the driver of subtle (∼3.1 pm/f.u.) atomic motions (teal blue arrows on atoms) that break the twofold symmetry in anhydrous ${\mathrm{LiInP}}_{2}S_{6}$, leading to a symmetry lowering and stabilization of the polar PG 3 in the hydrated phase. The polarization vector aligns along the *c*‐axis. A zoomed‐in view of the middle layer with atomic modulation in the fixed water reference frame is shown below. The anionic sulfur atoms move downwards relative to the cationic lithium, indium, and phosphorus atoms (bottom). (b) Temperature‐dependent (heating 30
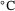
–250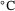, cooling back to RT) XRD patterns showing a transition at $∼\;160^{\circ }C$ from the hydrated ${\mathrm{LiInP}}_{2}S_{6}\cdot 3H_{2}O$ to the anhydrous ${\mathrm{LiInP}}_{2}S_{6}$. Temperature‐dependent XRD reveals reversible expansion of interlayer spacing upon hydration with diffraction peaks shifting to lower angles. (c) Relative energies after simulated annealing considering different structures for ${\mathrm{LiInP}}_{2}S_{6}\cdot 3H_{2}O$ indicating symmetry lowering from PG 32 (${\mathrm{LiInP}}_{2}S_{6}$) to PG 3 (${\mathrm{LiInP}}_{2}S_{6}\cdot 3H_{2}O$). (d) Total density of states (DOS) in units of states/eV and band structure of ${\mathrm{LiInP}}_{2}S_{6}$ for the anhydrous and hydrated phases. The band path is restricted to the in‐plane directions since there is little dispersion along the c‐axis of the trihydrate.

The ${\mathrm{LiInP}}_{2}S_{6}$ crystal structure readily accommodates water intercalation (Figure 1a) under ambient conditions at room temperature. To evaluate the resulting phases, temperature‐dependent XRD measurements were performed (Figure 1b). In XRD, at θ−2θ geometry, the specular reflections from the (000*l*) planes indicate that hydration leads to a change in the *c*‐axis lattice parameter from *c* = 13.02–19.70Å upon exposure to humid air. This arises from a dramatic expansion of the interlayer distance to 9.1Å (from 6.5Å), providing a clear evidence for water molecule intercalation between the vdW layers (see Figure S1). Temperature‐dependent measurements reveal that this enlarged c‐axis spacing due to hydration collapses to the anhydrous value upon dehydration occurring on heating above $∼160^{\circ }C$, confirming the reversible nature of the hydration process.

While single‐crystal XRD refinements fit equally well to both point groups 32 and 3, the predicted small lattice perturbation (∼3.1 pm/f.u.) lies near the detection threshold of conventional lab‐based diffraction, making it challenging to determine the correct symmetry unambiguously. Further, there exist identical systematic absences of diffraction peaks between the two space groups (*P* 312 and *P*3), which makes the evaluation of this structural transformation difficult to detect. This limitation motivates the need for a more sensitive probe of the picometer distortions leading to local symmetry change; Optical SHG described later will resolve this question in favor of the PG 3 for the hydrated phase.

As further evidence for this hydration‐driven transition, DSC identifies a sharp endothermic transition near 160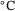, corresponding to the release of intercalated $H_{2}O$ from the vdW layers and the recovery of anhydrous ${\mathrm{LiInP}}_{2}S_{6}$ structure, consistent with the mass loss observed in thermogravimetric analysis (TGA) (Figure S2a). X‐ray photoelectron spectroscopy (XPS) shows unchanged elemental stoichiometry and core‐level binding energies upon hydration, indicating preserved chemical valence states. An additional O 1*s* feature at ∼ 533 eV, characteristic of hydrogen‐bonded molecular water, indicates interlayer water incorporation. Consistently, solid‐state 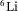 NMR measurements on hydrated and anhydrous ${\mathrm{LiInP}}_{2}S_{6}$ exhibit a single resonance near ∼1.07 ppm with no additional peak associated with hydrated ${\mathrm{Li}}^{+}$ species, indicating that the local Li environment remains largely unchanged upon hydration. Together, these results demonstrate that water intercalation primarily drives a lattice symmetry modification rather than altering the chemical valence states (Figure S2b and additional details in Supporting Information).

### 2.2 Microscopic Origin of Symmetry Lowering

To uncover the microscopic mechanism behind the symmetry lowering, we performed DFT calculations for both anhydrous and hydrated ${\mathrm{LiInP}}_{2}S_{6}$ structures. From Ref. [28], it is known that in ${\mathrm{LiInP}}_{2}S_{6}\cdot 3H_{2}O$, the water molecules occupy the vdW gap, forming hydrogen bonds with the sulfur atoms. Since the crystallographic details about water molecular configurations in the ${\mathrm{LiInP}}_{2}S_{6}\cdot 3H_{2}O$ phase were not resolvable via XRD, theoretical predictions of their configurations were performed using simulated annealing. Simulated annealing trials were repeated for three different symmetry constraints applied to $\mathrm{LiIn}P_{2}S_{6}$ layers (PG 32, PG 3, and unconstrained) while the water molecules were always unconstrained. For each symmetry constraint, the lowest energy structure across multiple simulated annealing trials was taken as the closest to the ground state.

Regardless of the symmetry of the ${\mathrm{LiInP}}_{2}S_{6}$ layers, the unconstrained water molecules were not found to adopt any symmetry, resulting in the overall ${\mathrm{LiInP}}_{2}S_{6}\cdot 3H_{2}O$ structure belonging to space group P1. The water molecules form hydrogen bonding chains with one bonding hydrogen and one non‐bonding hydrogen atom per water molecule. Two of the non‐bonding hydrogen atoms in the chain coordinate with ${\mathrm{LiInP}}_{2}S_{6}$ layers above and below. For more details, see the atomic coordinates provided in Table S4.

While our procedure is designed to produce realistic arrangements of water molecules, there may be higher symmetry arrangements in the form of longer periodicity configurations that could be accommodated by supercells of ${\mathrm{LiInP}}_{2}S_{6}\cdot 3H_{2}O$. It is also possible that, at room temperature, the water molecules are heavily disordered. A possibility that is supported by our simulated annealing procedure being able to be started at 300 K. Furthermore, the relative stability of intralayer versus water‐coordinated interlayer lithium was investigated, and it was found that interlayer lithium atoms coordinating with three water molecules are 1.168 eV lower in energy. We previously demonstrated interlayer cation exchange or cation transfer with other alkali cations in ${\mathrm{LiInP}}_{2}S_{6}$. Furthermore, cation transfer has been well studied in other metal‐${\mathrm{PS}}_{3}$ 2D materials [31], particularly by Clément and coworkers [32, 33, 34, 35] who demonstrated that intralayer metal cations can be exchanged for even large polyatomic interlayer cations. More generally, cation transfer is a feature of other layered metal sulfides, which we have previously reviewed in Ref. [36]. However, the layer exiting the reaction barrier of 417 meV provides a kinetic limitation (see Figure S16 for details). Overall, the relative formation energies of the ground states and SHG measurements corroborate the PG 3 assignment for the ${\mathrm{LiInP}}_{2}S_{6}$ layers in the ${\mathrm{LiInP}}_{2}S_{6}\cdot 3H_{2}O$ phase as seen in Figure 1c.

First principles calculations confirm that hydration‐driven polar lattice distortions break the parent 32 symmetry as shown in Figure 1a in ${\mathrm{LiInP}}_{2}S_{6}\cdot 3H_{2}O$. Applying the chiral $\Gamma_{1}^{-}$ mode (amplitude of 0.011 Å/f.u) to the centrosymmetric $\bar{3}2/m$ group (space group $P\bar{3}1c$) yields the subgroup 32 (space group P312). The PG 3 (space group P3) structure is a subgroup of PG 32 structure related by the polar $\Gamma_{2}^{-}$ mode (amplitude of 0.031 Å/f.u.).

The polar mode manifests in the *c*‐direction as opposing motion for the lithium and indium cations and their octahedral coordination shell of anionic sulfur atoms, as shown schematically in Figure 1a and Figure S4a. The sulfur atoms additionally move in the *a‐b* plane, widening and narrowing the triangular units they form. By modulating the combined (PG $\bar{3}$2/*m* → PG 3) mode, we find a parabolic potential energy landscape comprising a single minimum at a nonzero mode amplitude (see Figure S4b). This behavior indicates that water molecules bias the structure, lifting the degeneracy between the two bipolar states. We performed calculations of ${\mathrm{LiInP}}_{2}S_{6}$ layers and intercalated water molecules separately to further understand their interactions in Figure S15. The separated water layers have a high c‐axis polarization density of 1.0 $\mu {C/\mathrm{cm}}^{2}$. We estimated the magnitude of the electric field associated with this polarization density using the dipole‐layer correction required to counter it [37]. From this, we find that the water molecules impart a large unit cell average electric field of 9.80 MV/mm on the ${\mathrm{LiInP}}_{2}S_{6}$ layers.

We select the lowest energy ${\mathrm{LiInP}}_{2}S_{6}\cdot 3H_{2}O$ structure with PG 3‐constrained ${\mathrm{LiInP}}_{2}S_{6}$ layers as our representative structure for electronic and optical properties calculations since it has the lowest total energy of *all* structures predicted in the previous section, and ${\mathrm{LiInP}}_{2}S_{6}$ layers' symmetry matches the space group corresponding to the experimentally observed SHG tensor (discussed in the next section). The projected density of states (PDOS) and electronic band structure for ${\mathrm{LiInP}}_{2}S_{6}$ and ${\mathrm{LiInP}}_{2}S_{6}\cdot 3H_{2}O$ are shown together in Figure 1d where the band structure is restricted to in‐plane directions since the ${\mathrm{LiInP}}_{2}S_{6}\cdot 3H_{2}O$ band structure has negligible dispersion out of plane due to the widely separated layers, and the in‐plane lattice parameter, *a*, is similar between the ${\mathrm{LiInP}}_{2}S_{6}\cdot 3H_{2}O$ and ${\mathrm{LiInP}}_{2}S_{6}$ structures. The band structure shows that upon hydration, the lowest two conduction bands are associated with the empty In 5s states, which essentially become degenerate as the two ${\mathrm{LiInP}}_{2}S_{6}$ layers become electronically isolated. The effect is less dramatic in other bands. In ${\mathrm{LiInP}}_{2}S_{6}\cdot 3H_{2}O$, the isolated molecular orbitals of the water molecules form flat bands, the highest filled of which begin ∼ 0.5 eV below the valence band maximum (Figure 1d). The highest filled molecular orbital of water, the 1b_1_ orbital, predominantly comprises O 2p states as can be seen in the ${\mathrm{LiInP}}_{2}S_{6}\cdot 3H_{2}O$ PDOS.

### 2.3 Linear Optical Properties

**FIGURE 2:**
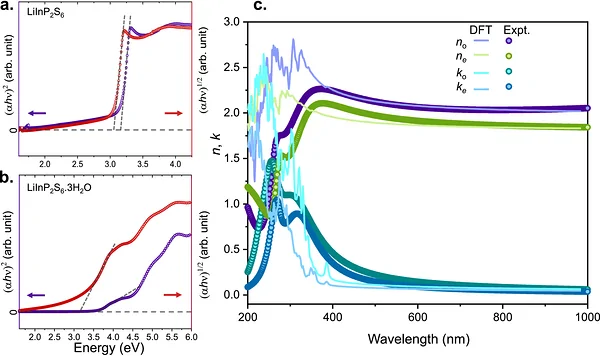
Tauc plots obtained from the UV–vis spectrum of (a) ${\mathrm{LiInP}}_{2}S_{6}$ and (b) ${\mathrm{LiInP}}_{2}S_{6}\cdot 3H_{2}O$ single crystals, considering indirect (red) and direct (purple) bandgaps. For ${\mathrm{LiInP}}_{2}S_{6}$ and ${\mathrm{LiInP}}_{2}S_{6}\cdot 3H_{2}O$, the indirect bandgaps are found to be ∼3 and ∼3.15 eV, respectively. (c) Measured (circles) and DFT calculated (solid lines) complex ordinary (*n* 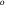, $k_{o}$) and extraordinary (*n* 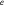, $k_{e}$) refractive indices for${\mathrm{LiInP}}_{2}S_{6}.3H_{2}O$.

Optical absorption measurements on ${\mathrm{LiInP}}_{2}S_{6}$ revealed an indirect gap of ∼3.00 eV and a direct gap of ∼3.15 eV (Figure 2a). When hydrated, ${\mathrm{LiInP}}_{2}S_{6}\cdot 3H_{2}O$ has an indirect bandgap of ∼3.15 eV and a direct gap of ∼3.40 eV (Figure 2b). The near‐ and mid‐infrared transparency windows were independently measured using UV–vis and FTIR spectroscopy (Figure S5a). Several absorption dips around ∼2.6, ∼2.94, ∼6.25, ∼8.57, and ∼9.80μm are seen arising from vibrational modes associated with molecular $H_{2}O$, including O–H stretching and bending modes. The infrared intensities of the Γ‐point vibrational modes were computed with DFT, and the spectrum can be seen in Figure S6 and tabulated in Table S2. A plot of the calculated vibrational mode intensities (Figure S6) is in reasonable agreement with the observed absorption bands in the 2–10 μm wavelength range (marked by red arrows).

The wavelength dispersion of the real and imaginary parts of the refractive index was measured using spectroscopic ellipsometry, as well as modeled with DFT (Figure 2c and Figure S5b). The index dispersion was parametrized with a Lorentz oscillator‐based model. The extracted oscillator parameters for the ordinary and extraordinary dielectric constants are reported in Table S3. The refractive indices obtained from first‐principles calculations are in excellent agreement with experiments, despite being evaluated within the independent‐particle approximation that is known to overestimate oscillator strengths and consequently the dielectric function due to the absence of local‐field effects. While the calculated spectra exhibit sharper features than the experimental data, reflecting the lack of finite‐temperature and lifetime broadening, the energies of the principal spectral features align well between theory and experiment. This overall agreement underscores the effectiveness of scissor‐shift‐corrected, computationally efficient DFT calculations for capturing the essential optical properties of the material.

### 2.4 Probing Hidden Symmetry Breaking Through Temperature‐Dependent SHG

**FIGURE 3:**
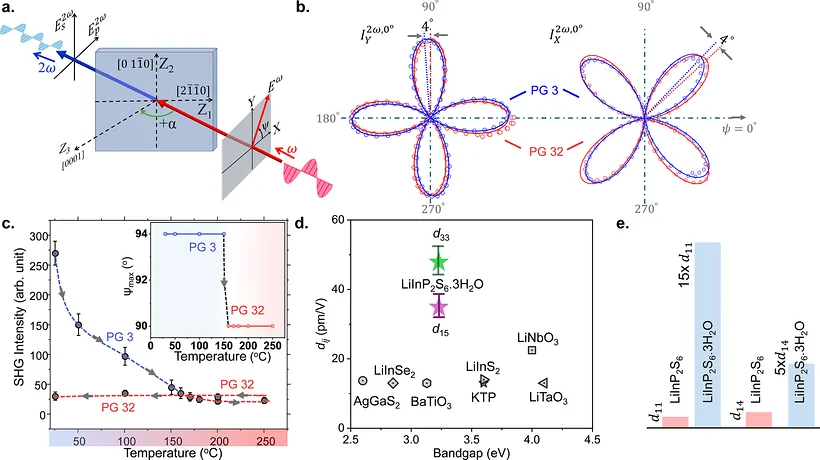
(a) Schematic of far‐field SHG polarimetry experimental geometry for 1550 nm to 775 nm SHG conversion. The crystallographic $Z_{1}$ $∥[2\bar{1}\bar{1}$0] and $Z_{2}$ $∥[01\bar{1}$0] were perpendicular to the crystal plane ($Z_{3}$ ∥[0001]). (b) Polarimetry plots of the SHG intensity as a function of the polarization of the incident light given by the polar angle, ψ, are shown in (left panel) $I_{Y}^{2\omega ,0}\left(\psi \right)$ for p‐polarized SHG and $I_{X}^{2\omega ,0}\left(\psi \right)$ for s‐polarized SHG (right panel) for normal incidence at RT (PG 3) and 160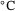 (PG 32). We observed a relative rotation of $∼4^{\circ }$ that arises in point group 3 (blue) about the [0001] axis compared to PG 32 (red), which again confirms the symmetry lowering in the hydrated phase. (c) Normal‐incidence SHG intensity versus temperature (heating 30
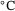
–250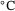, cooling back to RT) for ${\mathrm{LiInP}}_{2}S_{6}$ crystal. Bulk phase transition temperatures from a lower symmetry (3) → higher symmetry (32) are consistent with DSC and XRD measurements data, indicating symmetry‐lowering phase transition (32 →3) for ${\mathrm{LiInP}}_{2}S_{6}$ to ${\mathrm{LiInP}}_{2}S_{6}\cdot 3H_{2}O$ phase. T‐dependent SHG measurements show over an order‐of‐magnitude enhancement in the hydrated phase. Temperature‐dependent SHG intensity mirrors the structural transition seen in XRD and DSC. The inset shows that the relative rotation (at $\psi^{max}$) with T, which disappears once the temperature exceeds 160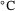, confirming the transition from the lower‐symmetry PG 3 phase to the higher‐symmetry PG 32 phase (marked by the color contrast of light blue and light red regions, respectively). (d) A comparison of the SHG coefficients of ${\mathrm{LiInP}}_{2}S_{6}\cdot 3H_{2}O$ with those of commercial NLO crystals [29, 30, 38, 39, 40, 41, 42, 43, 44]. Error bar represents the standard deviation of SHG coefficient of ($d_{33}$ and $d_{15}$) for ${\mathrm{LiInP}}_{2}S_{6}\cdot 3H_{2}O$ from the polarimetry fits for the nonresonant 1550–775 nm SHG conversion. (e) Nonlinear optical coefficients ($d_{11}$ and $d_{14}$) calculated from SHG modeling for ${\mathrm{LiInP}}_{2}S_{6}\cdot 3H_{2}O$ compared to ${\mathrm{LiInP}}_{2}S_{6}$, showing a large SHG enhancement as a result of symmetry lowering in ${\mathrm{LiInP}}_{2}S_{6}\cdot 3H_{2}O$ with water intercalation.

Hydration induces a structural transformation that conventional lab‐based diffraction failed to resolve, despite symmetry lowering due to emergent polar order during hydration. Optical SHG, a third‐rank polar tensor, offers the requisite sensitivity for observing this polar order. SHG polarimetry measurements were performed under far‐field geometry (see Figure 3a) with a beam diameter of ∼50 μm using a fundamental wavelength λ = 1.55 μm while detecting the SHG signal at λ/2 = 775 nm. It reveals the emergent polar order.

Figure 3b shows representative SHG polarimetry plots at room temperature and 160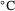, together with theoretical fits (solid lines) for point groups 3 and 32. For SHG polarimetry in ${\mathrm{LiInP}}_{2}S_{6}\cdot 3H_{2}O$ (Figure 3b), the best fit corresponds to the lower‐symmetry point group 3, while above $∼\;160^{\circ }C$‐corresponding to ${\mathrm{LiInP}}_{2}S_{6}$, the SHG polarimetry best fits with PG 32. The SHG polarimetry in normal incidence geometry, shown in Figure 3b indicated a relative rotation of the polar plots $∼\;4^{\circ }$ between the hydrated and anhydrous phases. This rotation angle, labeled as $\Delta \psi_{max}$ is plotted as a function of temperature in the inset of Figure 3c clearly showing a sharp transformation from point group 3 to 32 upon heating above $∼160^{\circ }C$. Theoretical fitting of the polarimetry data can account for this rotation in polar plots only by accounting for a point group change from 3 to 32, as discussed further below.

The SHG intensity $I^{2\omega }$ (at normal incidence), considering PG 32 can be written as $I_{Y,\mathrm{PG}32}^{2\omega }=A_{1}\;{\left|d_{11}\right|}^{2}\;\cos^{2}\left(2\psi \right)$ ,where $A_{1}$ is the prefactor, and maxima are at $\psi_{max,PG32}=\frac{k\pi }{2}\;,k\in Z$. Similarly, one can derive for the PG 3 $I_{Y,\mathrm{PG}3}^{2\omega }=A_{2}\;|\;d_{22}\cos\left(2\psi \right)+d_{11}\sin\left(2\psi \right)\;{|}^{2}=A_{2}\;R^{2}\sin^{2}\left(2\psi +\delta \right)$ where $R=\sqrt{d_{11}^{2}+d_{22}^{2}}$, $\delta =\arctan\;\left(\frac{d_{22}}{d_{11}}\right)$, and the maxima at $\psi_{\text{max,PG3}}=\frac{\pi }{4}-\frac{\delta }{2}+\frac{k\pi }{2}$ (see the Supporting Information and Figure S7 for detail). Inserting the values of $\frac{d_{22}}{d_{11}}$ from the fittings, we obtained $\Delta \psi_{\max}$ = $\psi_{max,PG3}-\psi_{max,PG32}$ = 4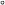. This further corroborates the hydration‐induced symmetry lowering from 32 to 3. These observations are fully consistent with DFT predictions of a polar distortion emerging upon water intercalation.

The temperature evolution of the SHG intensity (Figure 3c) reinforces this conclusion. On heating from 30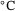 to 250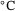, the SHG signal remains strong in the low‐symmetry polar phase (PG 3) and then drops sharply near 160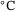 as the system transforms to the nonpolar PG 32 structure. The large SHG enhancement in PG 3 indicates that water intercalation not only modifies the interlayer structure but also induces spontaneous polarization through symmetry lowering. Upon cooling back to room temperature, the SHG intensity remains almost constant as the crystal remains dehydrated on measurement timescales. The transition temperature identified optically aligns closely with the DSC endotherm and the lattice change observed in XRD, confirming that the enhanced SHG originates from a hydration‐stabilized polar phase.

To further evaluate the environmental stability and reversibility of the hydration‐induced polar phase, time‐dependent SHG and humidity‐controlled measurements were performed (see Figures S11 and S12). The hydrated phase remains stable under ambient conditions over extended time periods (more than ∼ 4–5 weeks), while the SHG response can be reversibly recovered after thermal dehydration through rehydration under controlled humidity environments. From the phase recovery kinetics (Humidity dependent SHG measurement as shown in Figure S12) and estimation from FTIR water absorption peak area calculation (Figures S13 and S14), the recovery kinetics are strongly correlated to the water molecule content, with substantially faster restoration of the high‐SHG state under high relative humidity conditions. Additional details are provided in the Supporting Information.

### 2.5 Optical Second‐Harmonic Generation Tensor Determination

All SHG measurements were performed under far‐field geometry with a beam diameter of ∼50 μm using a fundamental wavelength of 1.55 μm, corresponding to a second‐harmonic wavelength of 0.775 μm. This ensures that both fundamental and SHG lie well below the bandgap of ∼ 3.2 eV, thus eliminating resonance and ensuring a nonresonant tensor measurement desired for laser applications.

To quantify the nonlinear coefficients and extract the complete second‐order susceptibility tensor, SHG polarimetry measurements were performed as a function of the incidence angle α (Figure 3a). The SHG polarimetry data were modeled for multiple α using a single set of tensor elements to ensure self‐consistency.

For the anhydrous ${\mathrm{LiInP}}_{2}S_{6}$ phase with PG 32, the allowed SHG tensor in Voigt notation is

(1) $$d_{ij}=\left(\begin{array}{cccccc} d_{11} & -d_{11} & 0 & d_{14} & 0 & 0\\ 0 & 0 & 0 & 0 & -d_{14} & -d_{11}\\ 0 & 0 & 0 & 0 & 0 & 0 \end{array}\right).$$

Only two independent coefficients, $d_{11}$ and $d_{14}$, are symmetry‐allowed. The fitting of independent sets of SHG polarimetry yields a ratio $d_{14}/d_{11}=-20$. Absolute magnitudes were determined by benchmarking the far‐field SHG intensity of ${\mathrm{LiInP}}_{2}S_{6}$ (at $\alpha =0^{\circ }$) against a z‐cut ${\mathrm{LiNbO}}_{3}$ reference under identical experimental conditions, giving $d_{11}=0.013$ pm $V^{-1}$ and $d_{14}=-0.26$ pm $V^{-1}$.

Upon hydration, ${\mathrm{LiInP}}_{2}S_{6}$ (${\mathrm{LiInP}}_{2}S_{6}\cdot 3H_{2}O$) undergoes a symmetry lowering from PG 32 to PG 3, introducing four additional independent SHG tensor elements. The tensor for PG 3 is expressed as

(2) $$d_{ij}=\left(\begin{array}{cccccc} d_{11} & -d_{11} & 0 & d_{14} & d_{15} & -d_{22}\\ -d_{22} & d_{22} & 0 & d_{15} & -d_{14} & -d_{11}\\ d_{31} & d_{31} & d_{33} & 0 & 0 & 0 \end{array}\right).$$

Here $d_{11}$, $d_{22}$, $d_{14}$, $d_{15}$, $d_{31}$, and $d_{33}$ are the six nonzero coefficients. Polarimetry fitting at multiple oblique incidence angles (also shown in Figure S10 in detail) has been performed using the Herman–Hayden formalism [45], as implemented in the open‐source #SHAARP software [46, 47].

**FIGURE 4:**
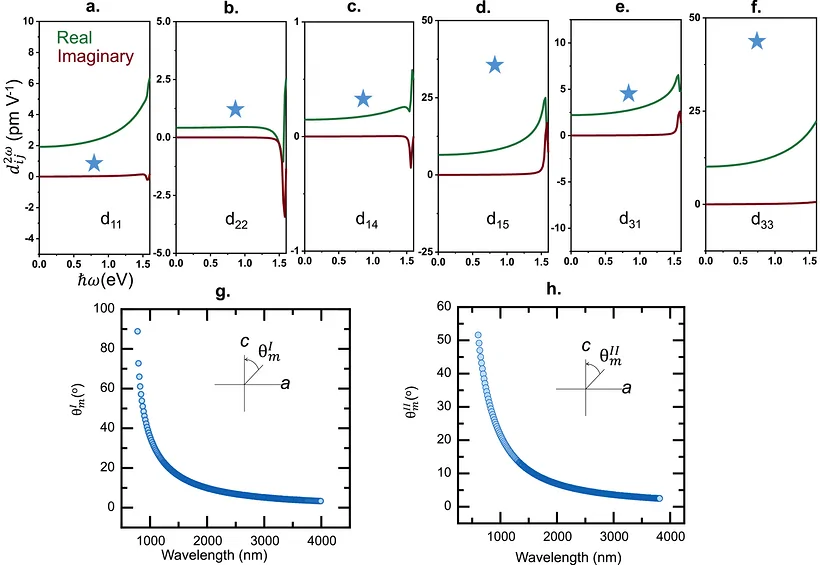
SHG coefficients and phase matching in ${\mathrm{LiInP}}_{2}S_{6}\cdot 3H_{2}O$: (a‐f) the variation of complex SHG coefficients ($d_{ij}$) as a function of energy (ℏω), calculated from first‐principles theory. Experimental values of $d_{ij}$ coefficients are marked with a blue star ($\lambda^{\omega }=1550$ nm generating nonresonant SHG at $\lambda^{\omega }=775$ nm). Phase‐matching angles for type‐I (g) and type‐II (h) configurations are plotted as a function of the fundamental wavelength (λ). The insets illustrate the phase‐matching angle in relation to the refractive index ellipsoids.

Absolute coefficients were again determined by referencing to z‐cut ${\mathrm{LiNbO}}_{3}$ (Figure S9). The dominant nonresonant tensor component in ${\mathrm{LiInP}}_{2}S_{6}\cdot 3H_{2}O$, $d_{33}\approx 47\;\text{pm}\;V^{-1}$, exceeds the benchmark values of state‐of‐the‐art infrared NLO crystals such as KTP, ${\mathrm{AgGaS}}_{2}$, ${\mathrm{BaTiO}}_{3}$, and ${\mathrm{LiInS}}_{2}$ (Figure 3d). Notably, hydration induces a colossal enhancement of the NLO response: $d_{11}$ increases by a factor of ∼15 and $d_{14}$ by ∼5 relative to the anhydrous phase (Figure 3e). The absolute values of $d_{ij}$ obtained from the global fits for PG 3 and PG 32 are listed in Table 1. A comparison between the experimentally extracted and theoretically calculated $d_{ij}$ is shown in Figure 4a–f.

**TABLE 1:** For LiInP_2_S_6_ (PG 32) and ${\mathrm{LiInP}}_{2}S_{6}\cdot 3H_{2}O$ (PG 3) single crystal, second‐order NLO tensor from experiments at 1.55μm fundemental wavelength.

Absolute values of $d_{ij}$ coefficients (pm/V)	PG 3	PG 32
$d_{11}$	0.198(0.25)	0.013(0.02)
$d_{22}$	1.52(0.2)	—
$d_{14}$	1.31(0.30)	−0.26(0.1)
$d_{15}$	35(5)	—
$d_{31}$	5.01(1.6)	—
$d_{33}$	47.1(7)	—

*Note*: Numbers within the parentheses are error bars representing the standard deviation of the SHG $d_{ij}$ coefficients.

Ab initio calculation of SHG tensor using the ${\mathrm{LiInP}}_{2}S_{6}\cdot 3H_{2}O$ with PG 3‐constrained ${\mathrm{LiInP}}_{2}S_{6}$ layers found from our simulated annealing procedure indicates that hydration enhances SHG response, but the lack of symmetry in the optically active water molecules leads to essentially all SHG tensor coefficients being active for this relaxed structure. In this calculation, the presence of water indirectly and directly affects the SHG response. The indirect effect is the aforementioned stabilization of the polar mode, which enhances SHG in the ${\mathrm{LiInP}}_{2}S_{6}$ layers by (1) activating SHG tensor coefficients by breaking symmetries that forbid them and (2) displacing atoms further from centrosymmetry. As a proxy measure for the latter point, the experimentally measured ${\mathrm{LiInP}}_{2}S_{6}$ structure and the PG 3 layers in the predicted ${\mathrm{LiInP}}_{2}S_{6}\cdot 3H_{2}O$ structure are, respectively, 0.019 Å/f.u. and 0.031 Å/f.u. distorted along the polar normal mode away from the centrosymmetric structure.

The direct effect of water on the SHG response comes from the addition of filled O 2p states high in valence band. The nonresonant excitation of these interlayer water states to the ${\mathrm{LiInP}}_{2}S_{6}$ conduction band states gives coupled SHG response contributions; this indicates that the net SHG response cannot be treated as a linear combination of separated ${\mathrm{LiInP}}_{2}S_{6}$ layers and interlayer water. To highlight this, SHG calculations were performed on the separated ${\mathrm{LiInP}}_{2}S_{6}$ layers and interlayer water as shown in Figure S15. Despite $d_{33}$ being the only significant SHG tensor component for interlayer water, SHG tensor for ${\mathrm{LiInP}}_{2}S_{6}\cdot 3H_{2}O$ is drastically changed from the SHG tensor of the ${\mathrm{LiInP}}_{2}S_{6}$ layers alone, with the magnitude of many tensor elements changing drastically (See in Figure S15e). This is generally a useful approach for computationally investigating the response of intercalating 2D materials.

### 2.6 Phase Matching and Applications

For efficient frequency conversion from fundamental to second harmonic, satisfying the phase‐matching condition is essential. Under ideal phase‐matching, the generated second‐harmonic waves interfere constructively, maximizing frequency conversion efficiency. For birefringent systems such as ${\mathrm{LiInP}}_{2}S_{6}$, two distinct phase‐matching schemes are possible: Type‐I and Type‐II. In Type‐I phase matching, the two generated SHG photons possess identical polarization states, whereas in type‐II, their polarizations are orthogonal. Given that ${\mathrm{LiInP}}_{2}S_{6}$ is a negative uniaxial crystal with the optic axis aligned along the crystallographic *c*‐axis, Type‐I phase matching can be realized with the fundamental electric field polarization at a specific phase‐matching angle, $\theta_{m}$, defined with respect to the optic axis (Figure 4g,h). The phase‐matching criteria are expressed as $n_{\omega }^{o}=n_{2\omega }^{e}\left(\theta_{m}\right)$ for type‐I and $n_{\omega }^{o}+n_{\omega }^{e}\left(\theta_{m}\right)=2n_{2\omega }^{e}\left(\theta_{m}\right)$ for type‐II. The refractive indices were modeled using the Cauchy relation in the transparent region above the band edge, enabling calculation of the corresponding phase‐matching angles for both cases (Figure 4g,h). These results indicate that ${\mathrm{LiInP}}_{2}S_{6}$ single crystals support both Type‐I and Type‐II phase matching in the nonresonant visible and infrared region. Although the calculated Type‐I and Type‐II phase‐matching angles suggest that birefringent phase matching should be accessible, direct experimental verification was not attempted in the present work because the available crystals are thin platelet‐like specimens that are difficult to cut and orient apprpoiopriately. Therefore, the calculated phase‐matching conditions are presented as predictions for future studies on larger, oriented, optically polished bulk crystals.

## 3 Conclusions

Typically moisture damage is an issue for commercial nonlinear optical crystals such as β‐${\mathrm{BaB}}_{2}O_{4}$, ${\mathrm{KH}}_{2}{\mathrm{PO}}_{4}$, ${\mathrm{KD}}_{2}{\mathrm{PO}}_{4}$, ${\mathrm{CsLiB}}_{6}O_{10}$ that are hygroscopic, degrading their optical performance. In contrast, hydration appears to be a powerful amplifier of the SHG performance in ${\mathrm{LiInP}}_{2}S_{6}$. Water intercalation in the nonpolar ${\mathrm{LiInP}}_{2}S_{6}$ (point group 32) is shown to activate the presence of a $\Gamma_{2}^{-}$ polar mode to create a hydrated ${\mathrm{LiInP}}_{2}S_{6}\cdot 3H_{2}O$ phase (point group 3). This phase exhibits a large nonresonant, phase‐matched SHG coefficient of $d_{33}∼47$ pm $V^{-1}$ at the telecommunications wavelength of 1550 nm, exceeding most well‐known optical crystals in the ∼3eV electronic bandgap range. It is also calculated from experimental index dispersion to be Type‐I and Type‐II SHG phase matchable. The process is reversible with hydration and dehydration, providing a powerful and simple chemical means to control the nonlinear optical response of this crystal. Density functional theory sheds light on both the phase transformation as well as the enhancement in the SHG. With improved control over the hydration process, novel applications could arise, such as on‐demand creation and erasure of optical circuit elements such as waveguides, and quasi‐phase‐matched devices across the visible and infrared wavelength regimes.

## 4 Methods

### 4.1 Synthesis and Crystal Growth

The following reagents: Lithium (99.9%, Sigma–Aldrich), indium droplets (99.99%, Thermo Scientific), phosphorus pentasulfide powder (99%, Sigma–Aldrich), and sulfur pieces (99.99%, Thermo Scientific) were used as received, without any additional purification. Lithium sulfide (${\mathrm{Li}}_{2}S$) was prepared following a modified literature protocol [27]. To synthesize ${\mathrm{LiInP}}_{2}S_{6}$, $P_{2}S_{5}$, In, S, and as‐synthesized ${\mathrm{Li}}_{2}S$ were mixed in their stoichiometric ratios and loaded into a carbon coated fused silica tube with inner and outer diameters of 14 and 18 mm, respectively. The tube was sealed under a 3.2 × 10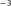 mbar vacuum. After sealing, the tube was placed in a synthesis furnace and gradually heated to 750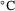 over 18 h. The sample was held at this temperature for 72 h, then slowly cooled to 300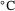 over 24 h, and finally cooled to room temperature over 12 h. ${\mathrm{LiInP}}_{2}S_{6}$ crystals were grown using the Chemical Vapor Transport (CVT) method in a two‐zone furnace. Pre‐synthesized polycrystalline ${\mathrm{LiInP}}_{2}S_{6}$ (2g) was mixed with iodine ($I_{2}$, 40 mg) as the transport agent and loaded into a silica tube with inner and outer diameters of 16 and 18 mm, respectively, and a length of 30 cm. The tube was then sealed under vacuum using an oxy/natural gas torch. The CVT process involved two temperature zones. In the source zone (650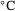), the loaded tube was first heated to 550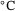 over 12 h and held there for 6 h. It was then heated to 650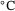 over 3 h and maintained at that temperature for 144 h before cooling down to room temperature over 12 h. In the deposition zone (550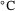), the opposite side of the tube was maintained at a lower temperature to facilitate crystal growth. The heating followed the same overall schedule: heating to 650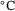 over 12 h, holding for 6 h, cooling to 550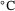 over 3 h, maintaining at 550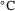 for 144 h, and finally cooling to room temperature over 12 h. High‐quality ${\mathrm{LiInP}}_{2}S_{6}$ crystals were collected from this zone.

### 4.2 Crystal Structure Characterization

X‐ray diffraction patterns were collected between 5.0
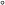
–70.0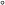 2θ‐ω with a step size of 0.017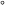 on a 240 mm radius Malvern Panalytical Empyrean x‐ray diffractometer equipped with a line source [Cu Kα 1‐2 (1.5405980/ 1.5444260Å)] x‐ray tube at 45.0 kV and 40.0 mA. The incident beam path included a BBHD optic with 0.04 radian Soller slits and a 1/8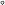 divergence slit. The diffracted beam path incorporated 0.04 radian Soller slits and a 0.5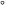 anti‐scatter slit. An X'Celerator detector operating in scanning line (1D) mode was used with an active length of 2.1223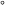 in 2θ. PHD lower and upper levels were set at 4.02 and 11.27 keV, respectively.

The sample was heated in an inert nitrogen environment using an Anton Paar HTK 1200N in situ oven. The sample temperature was calibrated using magnesium stabilized aluminum titanate, a material with anisotropic thermal expansion properties. In this method, the (250) and (002) diffraction peaks are measured, and Δ *d* (Δ *d* = *d*(250) − *d*(002)) is calculated and plotted as a function of temperature to create a calibration curve. The material and method were developed by Bryan Wheaton at Corning Inc. and have been validated via a round‐robin calibration study involving multiple labs.

### 4.3 Thermal Characterizations (DSC and TGA)

Differential scanning calorimetry (DSC) and thermogravimetric analysis (TGA) were simultaneously performed using a Discovery SDT 650 (TA ${\mathrm{Instruments}}^{\mathrm{TM}}$). Approximately 5 mg of hydrated ${\mathrm{LiInP}}_{2}S_{6}$ was placed in an alumina crucible and heated to 300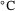 at a rate of 5 K $\min^{-1}$, followed by cooling to room temperature under uncontrolled forced convection. Measurements were conducted under a controlled nitrogen atmosphere. The number of water molecules (n = 3 in our case) associated with hydrated ${\mathrm{LiInP}}_{2}S_{6}$ was estimated using

$$n=\frac{M_{{\mathrm{LiInP}}_{2}S_{6}}\times \Delta m\%}{M_{H_{2}O}\times \left(1-\Delta m\%\right)}$$

where M denotes the molar mass of the respective compounds, and Δm% represents the percentage mass loss observed in the TGA curve.

### 4.4 X‐Ray Photoelectron Spectroscopy (XPS)

XPS measurements were carried out using a Physical Electronics VersaProbe III spectrometer equipped with a monochromatic Al Kα x‐ray source (hν=1486.6 eV) and a concentric hemispherical analyzer. Charge neutralization was achieved using both low‐energy electrons (<5 eV) and argon ions. The binding energy scale was calibrated using sputter‐cleaned Cu (Cu 2$p_{3/2}=932.62$ eV, Cu 3$p_{3/2}=75.1$ eV) and Au foils (Au 4$f_{7/2}=83.96$ eV). All peaks were referenced to the ${\mathrm{CH}}_{x}$ band in the C 1s spectrum at 284.8 eV. Measurements were performed at a takeoff angle of 45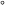 with respect to the sample surface, corresponding to an effective sampling depth of 3–6 nm (95% of the signal originating from this depth or shallower). Elemental quantification employed instrumental relative sensitivity factors (RSFs) accounting for x‐ray cross sections and inelastic mean free paths of the emitted electrons. For homogeneous samples, the standard deviation of major elements (>5 at.%) was typically <3%, while that of minor constituents could be higher. The analysis area was approximately 200 μm in diameter.

### 4.5 Linear Optical Properties

The linear optical properties of ${\mathrm{LiInP}}_{2}S_{6}$ single crystals were investigated using UV–vis spectroscopy (Agilent UV–VIS–NIR Spectrophotometer) and Fourier transform infrared (FTIR) spectroscopy (Bruker Vertex 80 with HgCdTe detector) in the spectral ranges of 0.25–2 and 2–10 μm, respectively. Both spectroscopic measurements were made by mounting the single‐crystal samples, with their crystallographic *c* direction facing the beam, onto the integrating sphere in transmission and reflection geometries. In FTIR, the data were compared with the reference spectrum in the air (for transmission) and gold (for reflection).Ellipsometry measurements were performed to determine the real and imaginary parts of the refractive index (*n* and *k*) within the 0.2–1 μm spectral range. Data were collected in two sample configurations, with the optic axis oriented both parallel and perpendicular to the plane of incidence. The data were simultaneously fitted to extract the corresponding oscillators, allowing the determination of *n* and *k* from the experimental parameters. Furthermore, the ordinary (*n* 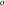) and extraordinary (*n* 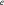) refractive indices were derived from the two experimental geometries.

### 4.6 SHG Polarimetry

SHG is a nonlinear optical process in which two photons at a fundamental frequency (ω) combine to generate a single photon at twice the frequency (2ω). In a noncentrosymmetric medium, the induced nonlinear polarization at frequency 2ω is given by

$$P_{i}^{2\omega }=d_{ijk}E_{j}^{\omega }E_{k}^{\omega },$$

where $d_{ijk}$ represents the second‐order nonlinear susceptibility tensor and $E_{j}^{\omega }$is the electric field inside the nonlinear medium at ω[12]. The indices i,j,k denote Cartesian polarization directions.

SHG polarimetry measurements were carried out in transmission geometry using a femtosecond laser (Spirit NOPA, 1550 nm fundamental wavelength, 100 fs pulse width, 1 MHz repetition rate). The transmitted second‐harmonic signal (2ω=775nm) was separated into its p‐ and s‐polarized components using an analyzer and detected by a photomultiplier tube. Both normal and oblique incidence conditions were investigated to fully probe the angular dependence of the SHG response.

The experimental geometry is illustrated in Figure 3b. Two crystallographic orientations were examined: $Z_{1}∥\left[2\bar{1}\bar{1}0\right]$ and $Z_{2}∥\left[01\bar{1}0\right]$, both lying perpendicular to the plane of incidence, with the optical axis defined as $Z_{3}∥\left[0001\right]$. For SHG polarimetry, the incident linearly polarized electric field was expressed as $E_{\omega }=\left(E_{\omega }\cos\psi ,E_{\omega }\sin\psi ,0\right)$, where ψ denotes the polarization angle relative to the laboratory X‐axis. The corresponding SHG intensity was recorded as a function of both ψ and the incidence angle α.

Quantitative analysis of the SHG tensor elements was performed by fitting the complete polarimetry datasets using the open‐source #SHAARP software package [46, 47], which implements the Herman–Hayden formalism for nonlinear optical modeling, including multiple reflections of both ordinary and extraordinary SHG waves within the crystal. This approach enabled a consistent extraction of the relative and absolute nonlinear coefficients for the ${\mathrm{LiInP}}_{2}S_{6}$ phases discussed in the main text.

### 4.7 Atomistic Simulations

All theoretical calculations were performed at the first‐principles level except for the simulated annealing of hydrated ${\mathrm{LiInP}}_{2}S_{6}$, which was performed with a machine‐learning interatomic potential (MLIP) before final optimization at the first‐principles level. First‐principles calculations were performed within the GPAW [48, 49] (version 25.1.1b1‐d55a5a3572) software package. The Atomic Simulation Environment [50] (ASE) (version 3.25.0b1‐eea53a3195) was used to perform simulated annealing, create plot band structures/band paths in the Brillouin zone (BZ), nudged elastic band (NEB) calculation, dimer method saddle point searching, and automate the general calculation workflow.

Ground state calculations and structure optimizations were performed using density functional theory (DFT) with the Perdew‐Burke‐Ernzerhof [51] (PBE) functional and its corresponding pseudopotential set included with GPAW (v0.9.20000). The self‐consistent (SCF) DFT calculations were performed with a planewave cut‐off of 600 eV, an augmentation grid with 2x the linear density of the planewave grid, a Γ‐centered 4×4×2 *k*‐point grid, a maximum real‐space grid spacing of 0.125 Å(for more accurate stresses), with an electronic eigenvalue convergence criterion of 10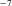 eV, and a charge density convergence criterion of 10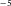 electrons per valence electron. DFT structure optimizations were terminated when no atomic force exceeded 5 meV/Å. For calculations of lithium migrating from intralayer to interlayer, 2 × 2 × 1 supercells were used to minimize periodic image interactions and a non‐Γ‐centered 2×2×1 *k*‐point grid was used to reduce the computational cost of the 152‐atom cell. The nudged elastic band (NEB) calculation used 16 interior images between the relaxed starting and ending structures. The dimer method was used to find the transition state for computing the migration barrier. The dimer search was initialized using the highest energy NEB image and the NEB tangent as the unstable mode guess. The dimer image separation distance was 0.001 Å, and the maximum number of mode rotations per position update was raised to 10 to ensure good convergence.

The configuration of the water molecules in the hydrated ${\mathrm{LiInP}}_{2}S_{6}$ structures was poorly resolved by x‐ray crystallography. To find the most probable, low‐energy configurations of the water molecules for subsequent first‐principles calculations, molecular dynamics‐based (MD) simulated annealing was performed with the small model variant of the MACE‐MP‐0b2 all‐element MLIP [52]. For each symmetry constraint applied to ${\mathrm{LiInP}}_{2}S_{6}$ sublattice, 32x simulated annealing trials were performed, varying the random number generator seed for randomized initial atomic velocities. For the initial structure, water molecules were arbitrarily inserted between anhydrous ${\mathrm{LiInP}}_{2}S_{6}$ layers after scaling the c lattice parameter to match those reported Ref. [28]. For the 2×2×1 supercell simulations with interlayer lithium, the initial structure was created by duplicating the lowest energy PG 3 structure in the *a* and *b* directions before a single lithium atom was displaced 5 Å along the *c*‐direction into the interlayer space. The lowest energy structure of 8x simulated annealing trials was used for subsequent calculations. Each simulated annealing trial consisted of three steps, (1) an NVT MD simulation with the thermostat target temperature linearly decreasing from 300 to 50 K over 3 ps, followed by (2) an MLIP‐based geometry optimization until no atomic force exceeded 0.1 meV/Å and finished with (3) DFT‐level structure optimization. The MD simulations were performed with ASE's Langevin NVT ensemble using a timestep of 1 fs, thermostatic friction parameter of 0.05 ${\mathrm{fs}}^{-1}$, and the ASE calculator object provided by the MACE software package [53, 54, 55]. Symmetry constraints were implemented by patching ASE's FixSymmetry class to allow application to selected subsets of atoms rather than globally. The patch is under review for future ASE releases. Vibrational mode analysis with corresponding IR intensities was performed using the two‐point central difference finite displacement method within ASE's Infrared module. The IR intensities are calculated from the finite differences of net polarization computed for each finite displacement step which was 0.01 Å.

First‐principles calculations of optical properties and atomic orbital projected density of states calculations and optical properties calculations were performed starting from non‐self‐consistent (NSCF) calculations where the linear *k*‐grid density was doubled for an 8×8×4 *k*‐grid. The high‐symmetry *k*‐path through the BZ used for the NSCF electronic band structure calculation follows the AFLOW standard [56]. For linear optical properties and SHG), the NSCF calculations were performed ensuring convergence of bands up 20 eV and 60 eV above the conduction band minimum, requiring 3.5× and 10× the number of bands in the ground state calculations, respectively. Calculation of the frequency‐dependent dielectric and SHG tensors [57, 58] was performed within the length‐gauge using the independent particle approximation (i.e., sum‐over‐bands approach) with an oscillator smearing, η, of 20 meV. Summations of the frequency‐dependent responses were restricted to the energy window of converged bands. A scissor shift of 1.252 eV for ${\mathrm{LiInP}}_{2}S_{6}$ and 0.946 eV for ${\mathrm{LiInP}}_{2}S_{6}\cdot 3H_{2}O$ was applied during optical calculations to correct for band gap underprediction by DFT. The scissor shift was kept the same for calculations of the ${\mathrm{LiInP}}_{2}S_{6}\cdot 3H_{2}O$ structure separated into ${\mathrm{LiInP}}_{2}S_{6}$ layers and interlayer water.

## Conflicts of Interest

The authors declare no conflicts of interest.

## Supporting information


**Supporting File**: adma74177‐sup‐0001‐SuppMat.pdf.

## Data Availability

The data that support the findings of this study are available on request from the corresponding author. The data are not publicly available due to privacy or ethical restrictions.
