# Supplementary material for: Reversible Hydration Tuning of Polar Order and Large Nonlinear Optical Response in a 2D van der Waals Crystal, LiInP2S6
Source: Adv Mater. 2026 Jul 31;38(52):e74177. doi: 10.1002/adma.74177 (PMC13579208; doi:10.1002/adma.74177)
Supplement: Supplementary file 1 [file ADMA-38-e74177-s001.pdf]

Supporting Information for "Reversible Hydration Tuning of Polar Order and Large Nonlinear Optical Response in a 2D van der Waals Crystal, $LiInP_2S_6$"

Jadupati Nag, Saugata Sarker*, Michael J. Waters*, Safdar Imam, Himirkanti Sarkar, James M. Rondinelli, Mercouri G. Kanatzidis, Venkatraman Gopalan* †

J. Nag, S. Sarker, H. Sarkar, A. Richardella, Venkatraman Gopalan
Department of Materials Science and Engineering, Pennsylvania State University, Millennium Science Complex Building University Park, PA 16802, USA
S. Imam, M.G. Kanatzidis
Department of Chemistry, Northwestern University, Evanston, Illinois 60208, United States
M.J. Waters, J.M. Rondinelli
Department of Materials Science and Engineering, Northwestern University, Evanston, Illinois 60208, United States
⋆ These authors have equal contributions.
† Corresponding author

The supporting information includes additional data supporting the XRD, DSC, XPS, FTIR, linear optical properties, SHG, and electronic structure.

1 XRD analysis

To distinguish between the room-temperature ($LiInP_2S_6.3H_2O$) and high-temperature ($LiInP_2S_6$) crystallographic phases, as shown in Figure S1a-b. Temperature-dependent XRD reveals reversible expansion of interlayer spacing from 6.45 Å to 9.1 Å upon H_2O uptake, with diffraction peaks shifting to lower angles as shown in Figure S1c. We summarize the expected X-ray diffraction extinction conditions for the relevant trigonal space groups. It is important to emphasize that systematic absences are governed by the space group symmetry (screw axes or glide planes), not by the point group alone. Extinction conditions (systematic absences) are specific sets of Miller indices (h, k, l) for which a crystal never produces a diffraction peak, even though the Bragg condition is satisfied.

Space group $P3$ (No. 143), point group 3:

Space group $P3$ is a primitive trigonal lattice containing a single pure three-fold rotation axis along the crystallographic c direction. Because it contains no screw axes and no glide planes, this space group imposes no systematic absences in X-ray diffraction.

Accordingly, all (h, k, l) reflections are symmetry-allowed, and any absent reflections arise solely from accidental structure-factor cancellation.

Space group $P312$ (No. 149), point group 32:

Space group $P312$ is a primitive trigonal space group characterized by a pure three-fold rotation axis along c and pure two-fold rotation axes perpendicular to c. Importantly, all symmetry elements are pure rotations.

As a result, $P312$ does not generate any systematic absences in X-ray diffraction. All reflections (h, k, l) are symmetry-allowed, and reflection conditions are identical to those of $P3$ from the standpoint of extinction rules.

Table S1: Symmetry elements and X-ray diffraction extinction conditions for the relevant trigonal space groups.

Space group	No.	Point group	Symmetry elements[a]	Systematic absences
$P3$	143	3	$3 \parallel c$	None
$P312$	149	32	$3 \parallel c$, $2 \perp c$	None

[a]Both space groups contain only *pure rotational* symmetry elements.

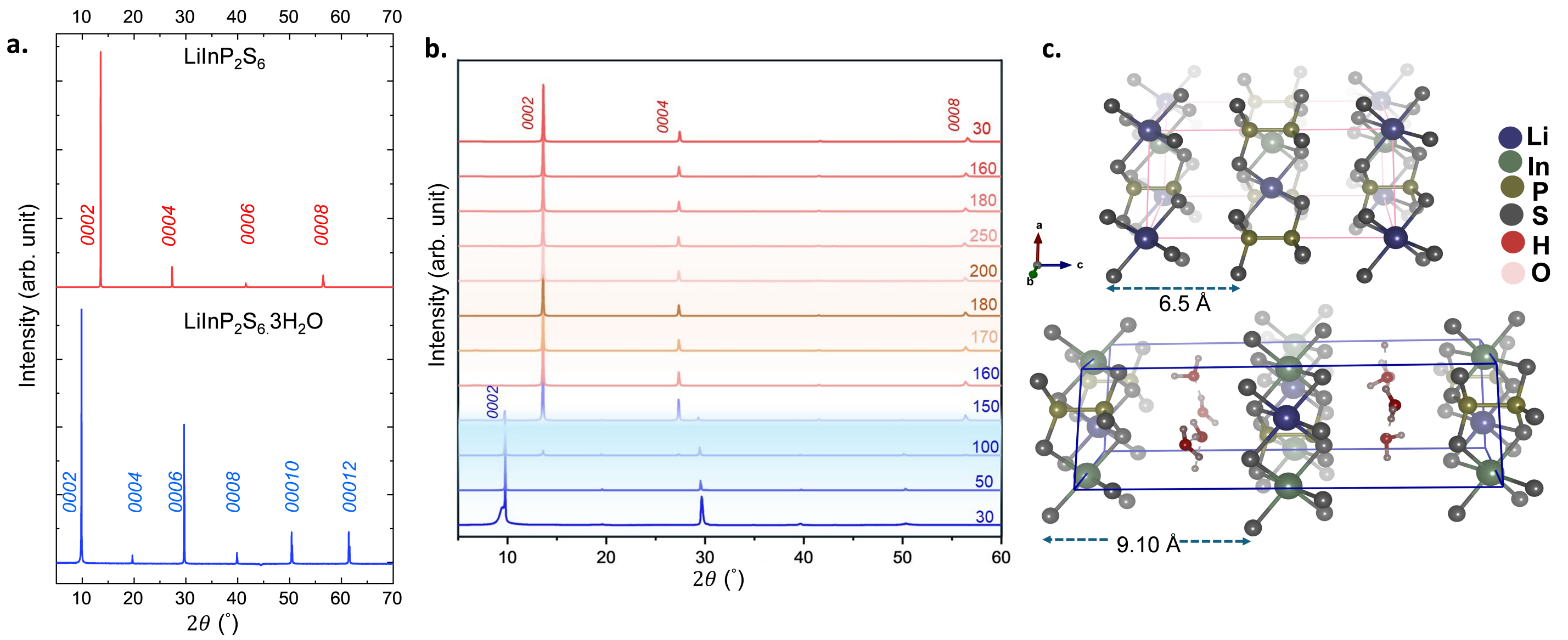

Figure S1: a. XRD pattern of $LiInP_2S_6$ and $LiInP_2S_6.3H_2O$ single crystals. b. Temperature-dependent XRD pattern of $LiInP_2S_6.3H_2O$ and $LiInP_2S_6$ single crystals. c. Crystal structure of $LiInP_2S_6$ (top) and hydrated $LiInP_2S_6$ (bottom) viewed perpendicular to b axis. Water molecules are intercalated in the interlayer space of $LiInP_2S_6$. Temperature-dependent XRD reveals reversible expansion of interlayer spacing from 6.45 Å to 9.1 Å upon H_2O uptake, with diffraction peaks shifting to lower angles.

Since both $P3$ (No. 143) and $P312$ (No. 149) contain only pure rotational symmetry elements, they impose *no systematic extinction conditions* in X-ray diffraction. Consequently, conventional XRD alone cannot unambiguously distinguish between the polar point group 3 and the higher-symmetry nonpolar point group 32 phases. Establishing the symmetry evolution between the anhydrous and hydrated phases, therefore, requires a tensor-sensitive probe, such as optical second-harmonic generation (SHG), which directly correlates point-group symmetry through its nonlinear susceptibility tensor elements.

2 DSC and XPS

DSC measurements were performed to examine the structural transition and quantify the extent of water intercalation in the vdW layers. DSC revealed distinct endothermic events near 160 °C, indicative of a first-order phase transition. This transition is chemically attributed to the release of intercalated water molecules into the atmosphere, resulting in a structural modification (becoming pristine $LiInP_2S_6$). Thermogravimetric Analysis (TGA) further supports this interpretation, showing a clear mass loss associated with dehydration (see Figure S2a). Analyzing TGA data, the chemical formula of the hydrated compound was estimated to be $LiInP_2S_6.3H_2O$ (see Experimental Section). Temperature-dependent XRD and DSC consistently reveal a sharp phase transition and a water uptake of 3 H_2O, indicating a stable $LiInP_2S_6.3H_2O$ phase with minimal compositional fluctuation under ambient conditions.
To probe the local bonding environment and electronic states, X-ray photoelectron spectroscopy (XPS) was employed (Figure S2b) for both $LiInP_2S_6$ and $LiInP_2S_6.3H_2O$. The survey spectra reveal all constituent core-levels corresponding to Li $1s$, In $3d_{5/2}$, P $2p$, and S $2p$, confirming the chemical composition of the compound in both anhydrous and hydrated phases. Quantitative analysis indicates that the elemental ratios remain close to the nominal stoichiometry within the experimental uncertainty, suggesting that water intercalation does not alter the bulk chemical composition. Additionally, O $1s$ XPS is sensitive to the local chemical environment and can distinguish between different water states. In our case, the hydrated $LiInP_2S_6.3H_2O$ exhibits an O $1s$ peak centered around ∼533 eV as shown in Figure S3a, which is characteristic of molecular or weakly bound hydrogen-bonded water. In contrast, strongly alkali-ion-coordinated (hydrated) water typically appears at lower binding energies due to enhanced electronic screening and charge redistribution around the oxygen atom. This assignment is consistent with prior XPS studies of water in different chemical environments. Furthermore, infrared spectroscopy (discussed in Section 5) con-

firms the presence of water in $LiInP_2S_6{\cdot}3H_2O$. The hydrated phase exhibits a broad O-H stretching band at ~3400-3700 cm^{-1} (~2.94-2.7 μm) and an H-O-H bending mode near ~1600 cm^{-1} (~6.25 μm), consistent with hydrogen-bonded molecular water. The preservation of stoichiometry together with the consistent core-level binding energies evidences that hydration primarily affects the lattice symmetry rather than the elemental valence states.

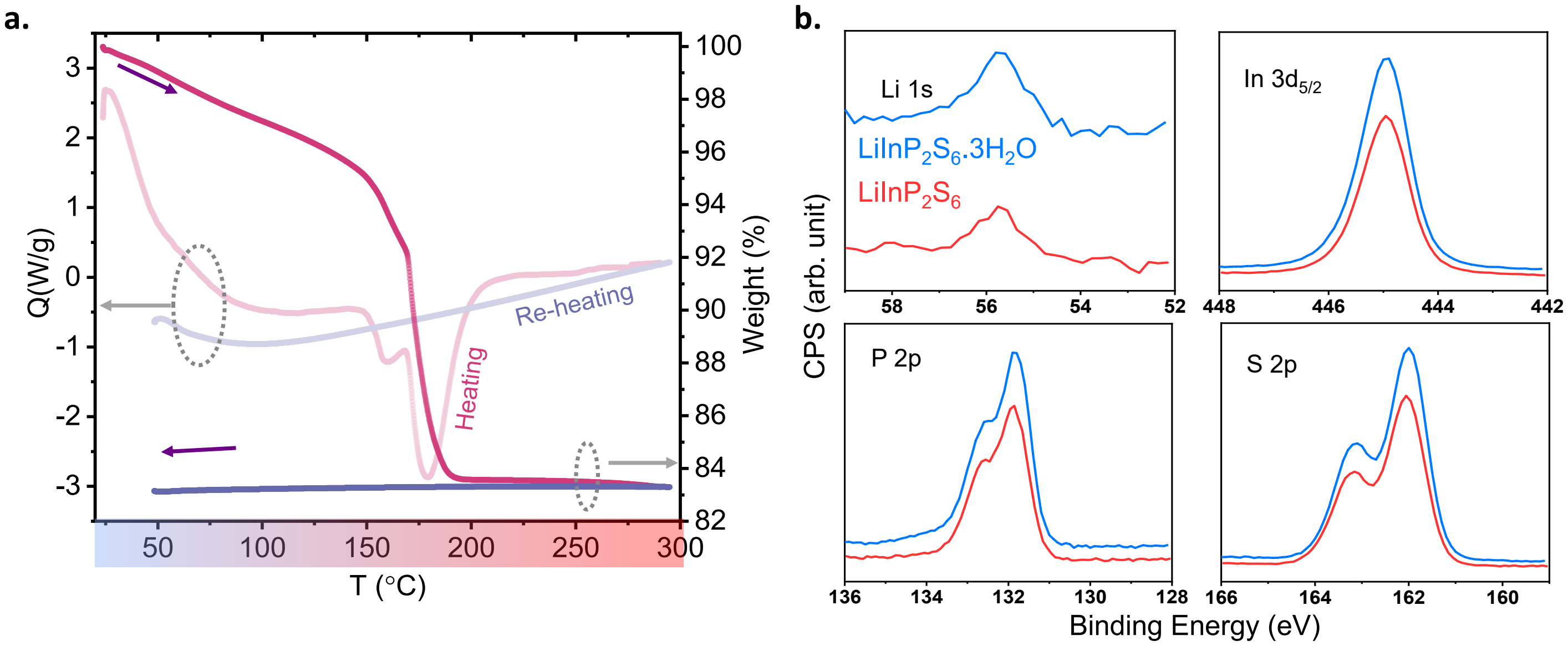

Figure S2: (a) Normalized heat flow Q (DSC) and weight loss (TGA) as a function of temperature for $LiInP_2S_6.3H_2O$ (heating) and $LiInP_2S_6$ (re-heating). (b) Core level XPS spectra for Li 1*s*, In $3d_{5/2}$, P 2*p*, and S 2*p* for $LiInP_2S_6$ (red) and $LiInP_2S_6.3H_2O$ (blue).

3 NMR

Solid-state ^{6}Li NMR measurements were performed on a Bruker TopSpin 3.7.0 system using a one-pulse direct-excitation pulse program. The observed nucleus was ^{6}Li, with a resonance frequency of 73.562182 MHz, corresponding to a 500 MHz-class spectrometer. The experiment was carried out under magic-angle spinning at 5 kHz using a Bruker MAS probe. The acquisition used 2048 scans, 4 dummy scans, a recycle delay of 5s, and a ^{6}Li excitation pulse length of 1.3μs. The spectral width was 36.765kHz, with 4096 acquired points. Proton decoupling was not used during acquisition, as both the decoupler nucleus and decoupler blanking nucleus were set to off. The nominal acquisition temperature was approximately 298K. To further examine the possibility of hydrated interlayer Li^+ species, solid-state ^{6}Li NMR measurements were performed on both hydrated $LiInP_2S_6{\cdot}3H_2O$ and anhydrous $LiInP_2S_6$ samples. As shown in Figure S3b, both spectra exhibit a single dominant resonance centered near ~1.07 ppm with no additional resolved features within the experimental detection limit. The absence of a distinct shifted resonance upon hydration indicates that Li predominantly retains a similar local chemical environment in both phases, rather than forming a separate hydrated interlayer Li^+ configuration. These results are consistent with the O 1*s* XPS and infrared spectroscopy measurements, which support the presence of intercalated molecular/weakly hydrogen-bonded water instead of strongly Li^+-coordinated water.

4 Theoretical Calculations

For the 32 and 3 equilibrium structures, the $LiInP_2S_6$ layers were found to be 0.011 Å/f.u. and 0.031 Å/f.u. from the centrosymmetric $\bar{3}2/m$ symmetry. Applying the chiral Γ_1^- mode to the centrosymmetric $\bar{3}2/m$ group (space group $P\bar{3}1c$) yields the subgroup 32 (space group $P312$). The PG 3 (space group $P3$) structure is a subgroup of PG 32 structure related by the polar Γ_2^- mode. The polar mode manifests in the *c*-direction as opposing motion for the lithium and indium cations and their octahedral coordination

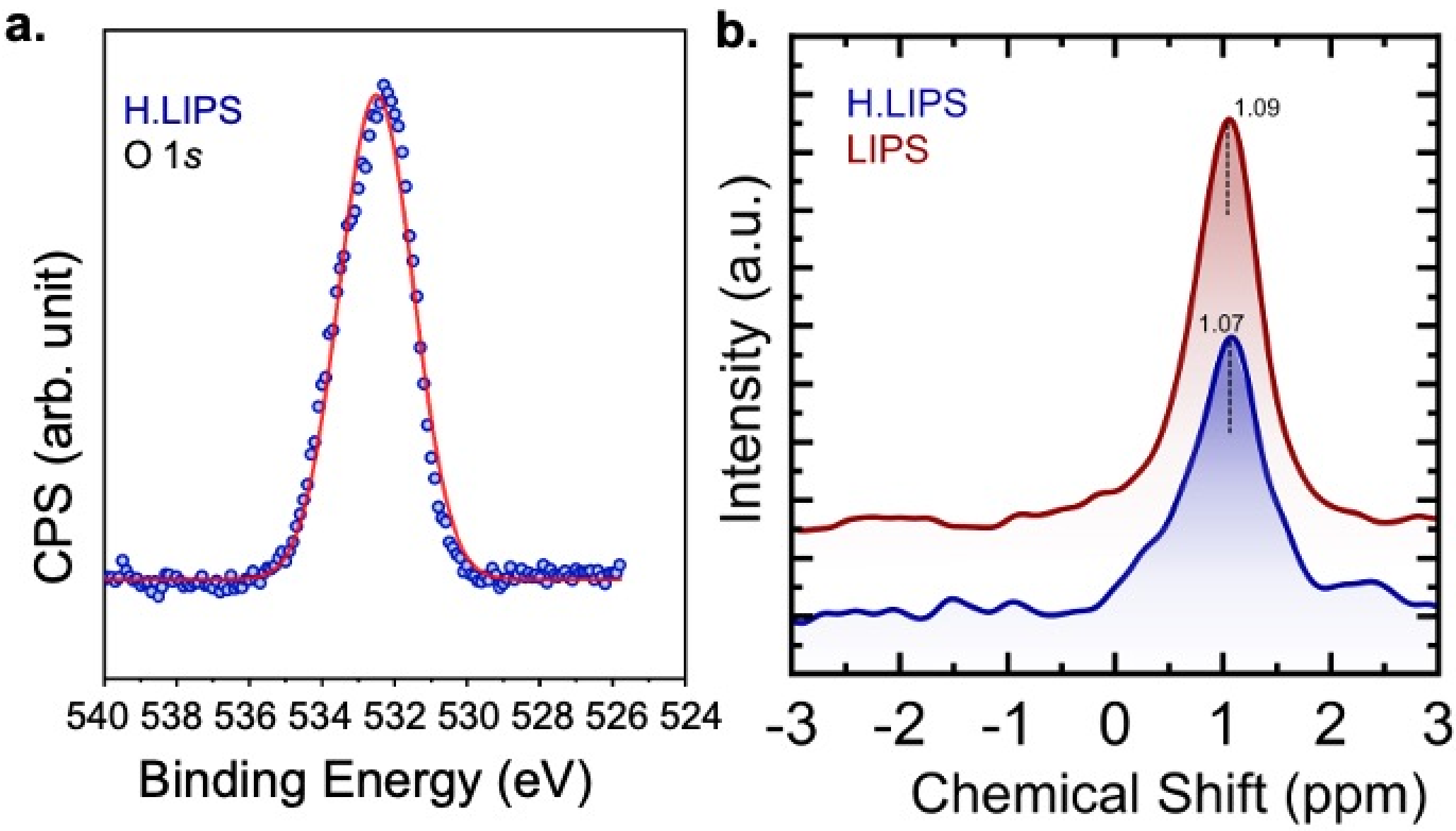

Figure S3: (a) O 1s XPS peak around 533 eV of $LiInP_2S_6.3H_2O$ along with peak fitting (Voigt function) (b) Solid-state ^6Li NMR spectra of anhydrous $LiInP_2S_6$ and hydrated $LiInP_2S_6{\cdot}3H_2O$ measured at room temperature. Both spectra exhibit a single dominant resonance centered near ∼1.07 ppm with no additional resolved peak associated with hydrated Li^+ species, indicating that the local Li environment remains largely unchanged upon hydration.

shell of anionic sulfur atoms, as shown schematically in Figure S4a. The sulfur atoms additionally move in the a-b plane, widening and narrowing the triangular units they form. By modulating the combined (PG $\bar{3}2/m \rightarrow$ PG 3) mode, we find a parabolic potential energy landscape comprising a single minimum at a nonzero mode amplitude (Figure S4b). This behavior indicates that water molecules bias the structure, lifting the degeneracy between the two bipolar states.

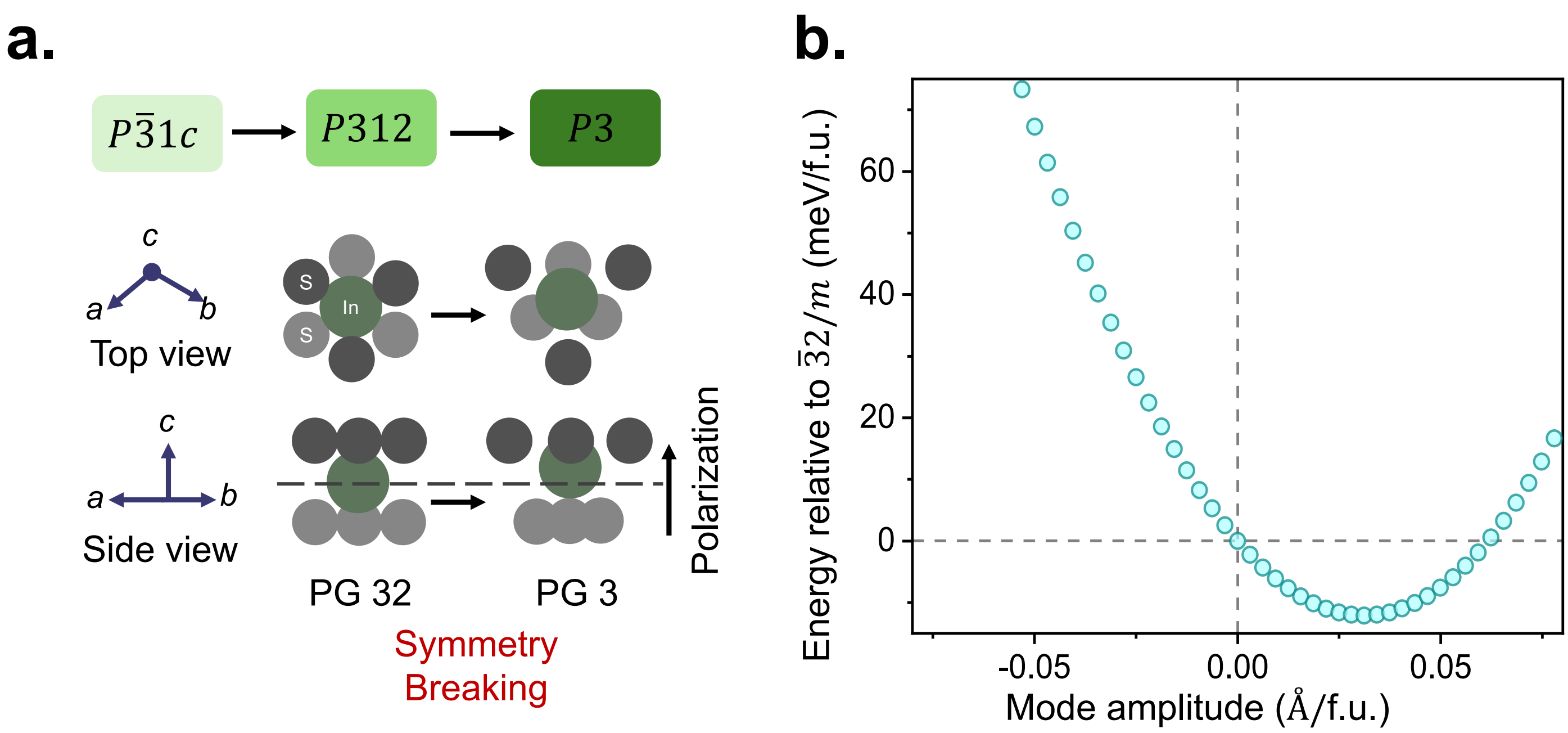

Figure S4: (a) Schematic of symmetry breaking from PG 32 → PG 3, ($D_3 \rightarrow C_3$). The three 2-fold axes ⊥c are broken by hydration, which results in a polarization $P \parallel c$, while the 3-fold axis survives. (b) Modulation of displacement mode from $\bar{3}2/m$ to 3 minimum.

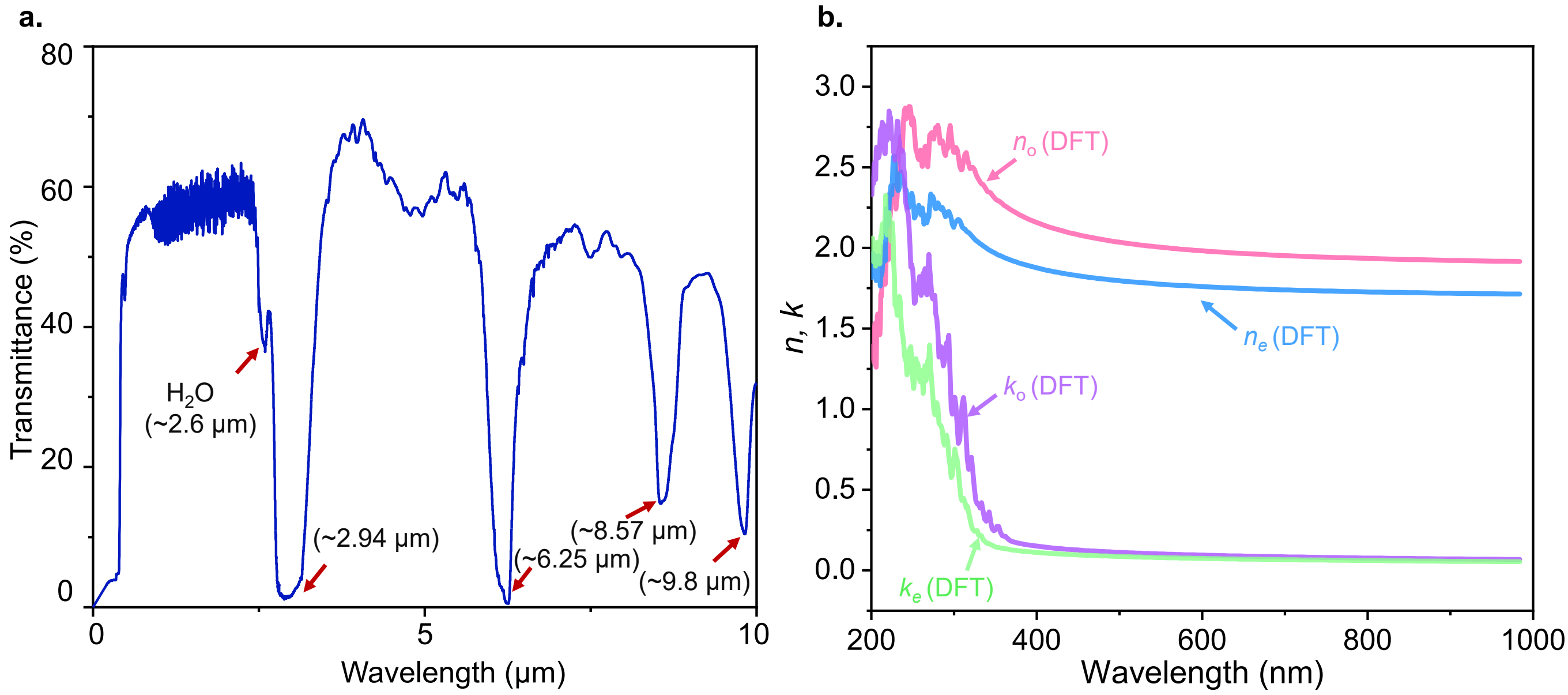

Figure S5: a. The transmission spectrum of the $LiInP_2S_6.3H_2O$ single crystal in the near-infrared (NIR) and mid-infrared (MIR) ranges, which indicates a broad transparency range of 1.25 to 10 μm. The red arrows near the absorption dips denote the presence of H_2O. b. DFT calculated (solid lines) ordinary (n_0) and extraordinary (n_e) refractive indices for $LiInP_2S_6$.

5 Linear optical properties

We first characterize the linear optical response of the crystal. Optical absorption spectroscopy conducted on the $LiInP_2S_6$ and $LiInP_2S_6.3H_2O$ crystals indicate indirect optical bandgap of ∼3.0 eV and ∼3.15 eV, respectively, as shown in the manuscript. The near- and mid-infrared transparency windows were independently measured using UV–Vis and FTIR spectroscopy (Figure S5a). Several narrow absorption dips are observed and are highlighted by red arrows. These features are attributed to vibrational modes associated with molecular H_2O, including O-H stretching and bending modes. These well-defined water-related absorption features support water intercalation in $LiInP_2S_6.3H_2O$. The single crystal is optically transparent from approximately 1 μm to beyond 10 μm in wavelength, except for the localized water-related absorption features. When both the fundamental and second-harmonic wavelengths lie within this transparency window, optical absorption is negligible; measurements performed under these conditions (for our case, fundamental wavelength ω= 1.55 μm (2ω=0.775 μm)) are therefore referred to as non-resonant SHG, which we discuss next.

The real and imaginary parts of the refractive index for $LiInP_2S_6$ from the theoretical calculations are shown in Figure S5b. The experimental ellipsometry data (shown in the manuscript) have been fitted with oscillator-based modeling to refine the real and imaginary parts of the dielectric constant at the optical frequency. We utilized a Lorentz oscillator to define the response of a system under driven optical oscillation. The oscillator can be defined as,

$$L(E) = \frac{A\gamma^2}{(E - E_0)^2 + \gamma^2}$$

Here, A is the amplitude of the oscillator, E is the energy, γ is the broadening parameter, and E_0 is the center energy in eV. The parameters for the ordinary and extraordinary dielectric constants are reported in Table S3.

The refractive indices obtained from first-principles calculations show excellent agreement with experiment, despite being evaluated within the independent-particle approximation, which is known to overestimate oscillator strengths and consequently the dielectric function, due to the absence of local-field effects. While the calculated spectra exhibit sharper features than the experimental data, reflecting the lack of finite-temperature and lifetime broadening, the energies of the principal spectral features align

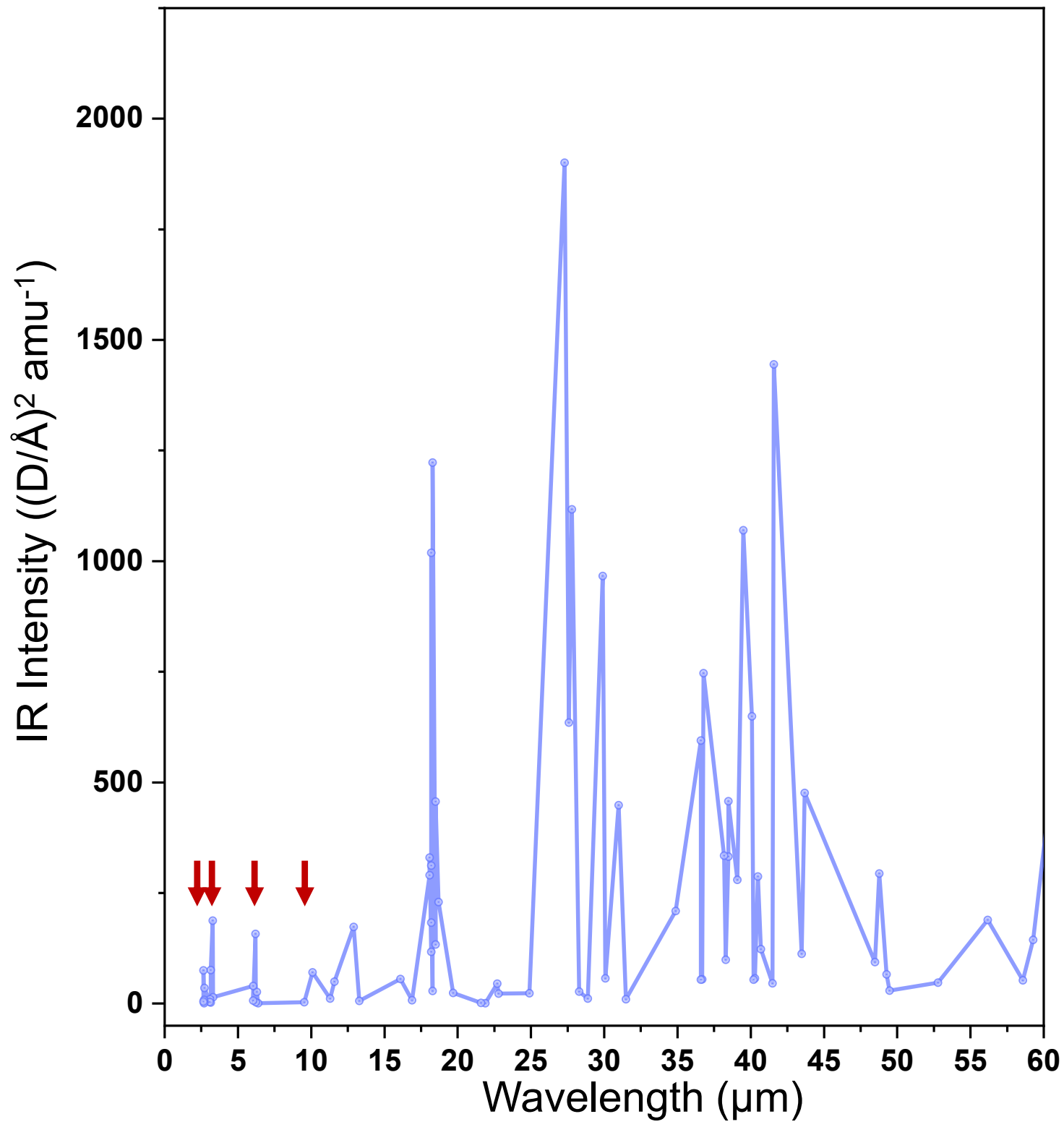

Figure S6: IR Spectrum calculated for the lowest energy $LiInP_2S_6.3H_2O$ structure, where the $LiInP_2S_6$ layers were constrained to PG 3. The calculated vibrational mode intensities are in reasonable agreement with the observed absorption bands in the 2-10 μm wavelength range (marked by red arrows).

well between theory and experiment. This overall agreement underscores the effectiveness of scissor-shift–corrected, computationally efficient DFT calculations for capturing the essential optical properties of the material.
The infrared-active Γ-point vibrational modes were computed using DFT, with the resulting spectrum shown in Figure S6 and listed in Table S2. The calculated mode intensities agree reasonably well with the experimentally observed absorption bands (Figure S5a) in the 2–10 μm range (red arrows).

6 SHG Analysis

Second-harmonic generation (SHG) is a nonlinear optical process arising from electric-dipole interactions and occurs only in noncentrosymmetric materials that lack inversion symmetry. As a result, SHG serves as a highly sensitive probe of crystal symmetry. In SHG polarimetry measurements, the SHG intensity was recorded as a function of the polarization angle ψ of the incident linearly polarized fundamental light, with the electric field given by

$$E^{\omega} = (E^{\omega}\cos\psi,\ E^{\omega}\sin\psi,\ 0)\,.$$

The SHG intensity $I^{2\omega}$ (at normal incidence, $\alpha = 0^o$) for PG 3 can be written as:

$$\begin{aligned} I^{2\omega}_{X,0^\circ} &\propto \Big(A_1\, d_{22} \cos^2\psi + A_2\, d_{11} \cos\psi\, \sin\psi - A_3\, d_{22} \sin^2\psi\Big)^2, \\ I^{2\omega}_{Y,0^\circ} &\propto \Big(A_4\, d_{11} \cos^2\psi - A_5\, d_{22} \cos\psi\, \sin\psi - A_6\, d_{11} \sin^2\psi\Big)^2. \end{aligned} \tag{1}$$

The SHG intensity at any oblique incidence (α) for PG 3 can be written as:

$$\begin{aligned} I^{2\omega}_{X,\alpha} \propto \Big(&A_7\, d_{15} \cos^2\psi + A_8\, d_{22} \cos^2\psi + A_9\, d_{31} \cos^2\psi + A_{10}\, d_{33} \cos^2\psi \\ &+ A_{11}\, d_{11} \cos\psi\, \sin\psi + A_{12}\, d_{14} \cos\psi\, \sin\psi - A_{13}\, d_{22} \sin^2\psi + A_{14}\, d_{31} \sin^2\psi\Big)^2, \end{aligned} \tag{2}$$

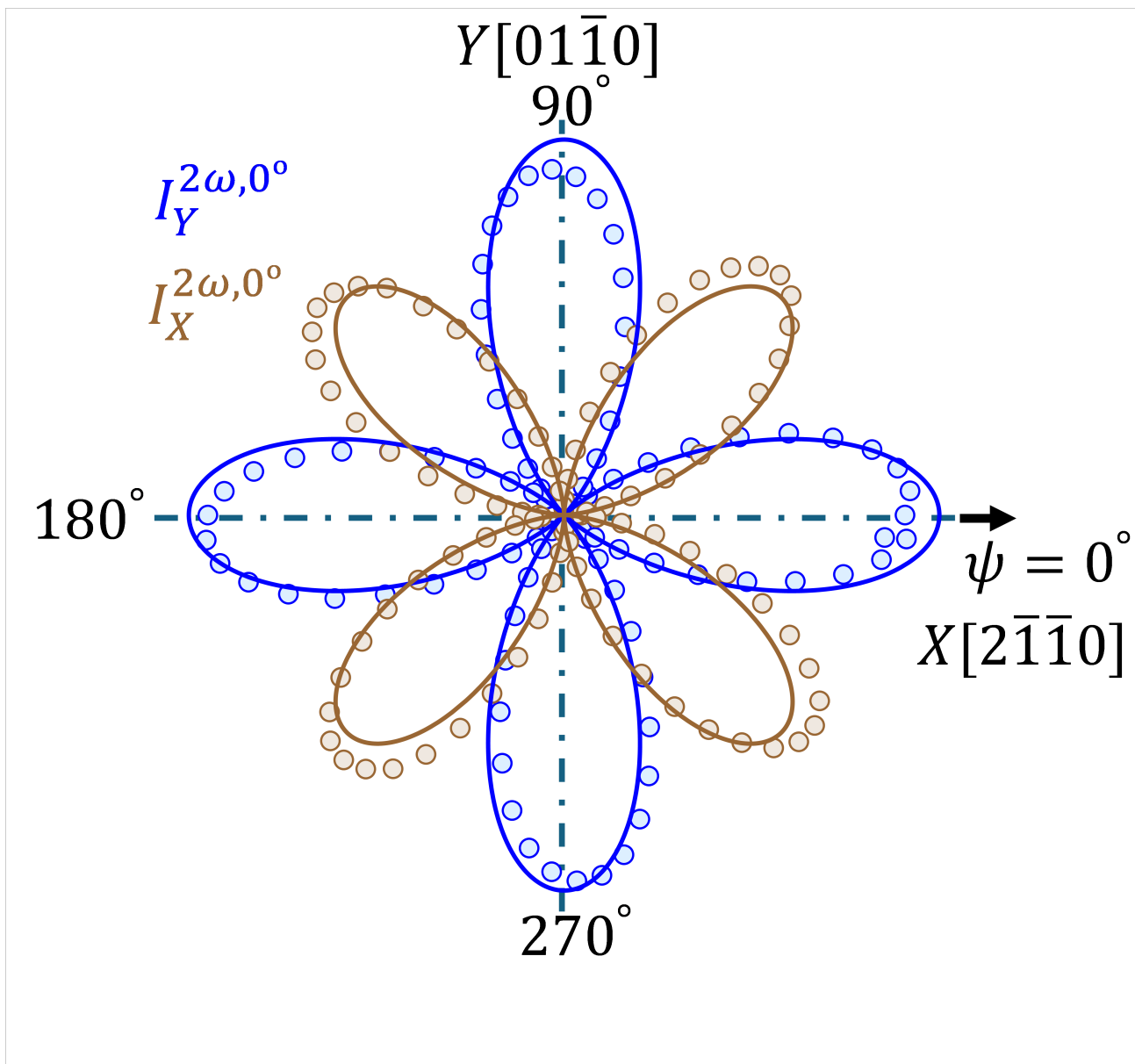

Figure S7: For $LiInP_2S_6.3H_2O$, normal-incidence SHG polarimetry (symbols) fitted under a forced PG 32 constraint (solid lines) exhibits significant deviations and poor fitting statistics, demonstrating the inadequacy of PG 32 symmetry for the hydrated phase.

$$I_{Y,\alpha}^{2\omega} \propto \Big(A_{15}\, d_{11} \cos^2\psi - A_{16}\, d_{14} \cos^2\psi + A_{17}\, d_{15} \cos\psi\, \sin\psi - A_{18}\, d_{22} \cos\psi\, \sin\psi - A_{19}\, d_{11} \sin^2\psi\Big)^2. \tag{3}$$

The SHG intensity $I^{2\omega}$ (at normal incidence, $\alpha = 0°$) for PG 32 can be written as:

$$\begin{aligned} I_{X,0°}^{2\omega} &\propto A_1\, d_{11} \cos\psi\, \sin\psi, \\ I_{Y,0°}^{2\omega} &\propto A_2\, d_{11} \cos^2\psi + A_3\, d_{11} \sin^2\psi. \end{aligned} \tag{4}$$

The SHG intensity at any oblique incidence (α) for PG 32 can be written as:

$$I_{X,\alpha}^{2\omega} \propto A_4\, d_{11} \cos\psi\, \sin\psi + A_5\, d_{14} \cos\psi\, \sin\psi. \tag{5}$$

$$I_{Y,\alpha}^{2\omega} \propto A_6\, d_{11} \cos^2\psi + A_7\, d_{14} \cos^2\psi + A_8\, d_{11} \sin^2\psi. \tag{6}$$

Here, A_i are the prefactor constants.
For $LiInP_2S_6.3H_2O$, the SHG polarimetry data (symbols) acquired at normal incidence cannot be satisfactorily described when the fitting is constrained to the PG 32 symmetry, as evidenced by pronounced deviations between the experimental data and the model fits (solid lines) as shown in Figure S7. This mismatch indicates that the PG 32 tensor formalism fails to capture the symmetry of the hydrated phase.

SHG polarimetry measurements reveal a small relative rotation of $\sim 4°$, as discussed in the main text (Fig. 3b). This rotational offset is a symmetry-specific feature that is allowed only for point group 3 and is forbidden in point group 32 (see Figure S8). Its observation, therefore, provides direct evidence for hydration-induced symmetry lowering. To elucidate the origin of this relative rotation, we explicitly write down the SHG intensity expressions for both point groups below. For PG 32, the SHG intensity at normal incidence can be written as:

$$I_{Y,PG32}^{2\omega} = A\, |d_{11}|^2 \cos^2(2\psi)$$

Maxima:

$$\psi = \frac{k\pi}{2}, \quad k \in \mathbb{Z}.$$

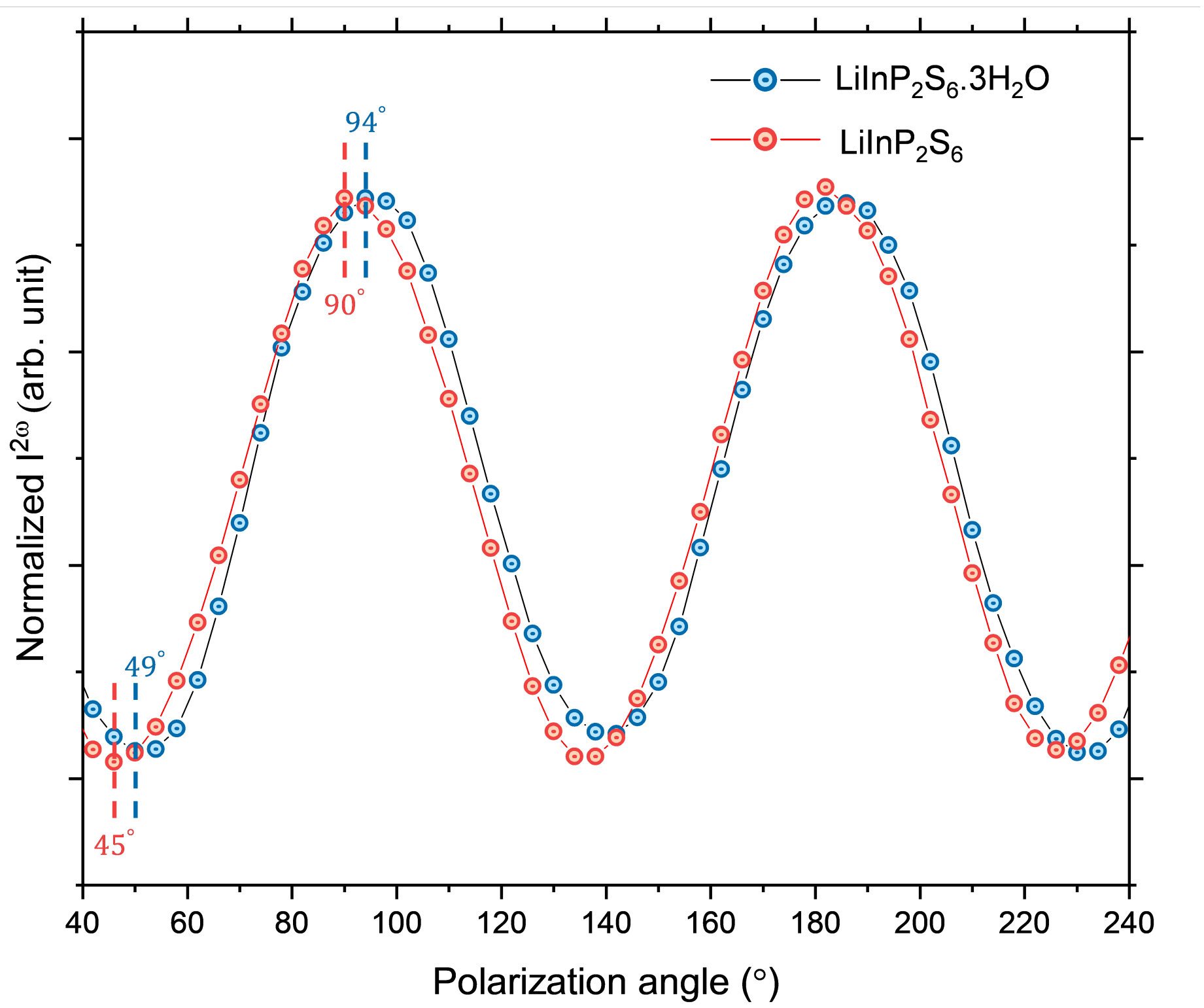

Figure S8: Normalized SHG intensity for normal incidence for $LiInP_2S_6.3H_2O$ (PG 3) at RT and for $LiInP_2S_6$ (PG 32) at 160 °C. We observed a relative rotation of $\sim 4°$ that arises in point group 3 (red) about the [0001] axis compared to PG 32 (blue). ψ^{max} for PG 32 is 90 °, while for PG 3 it is 94 °, which again confirms the symmetry lowering in the hydrated phase.

Minima:

$$\psi = \frac{\pi}{4} + \frac{k\pi}{2}, \quad k \in \mathbb{Z}.$$

The SHG intensity for PG 3:

$$I^{2\omega}_{Y,PG3} = A\left(d_{22}\cos 2\psi + d_{11}\sin 2\psi\right)^2,$$

where $A > 0$ and $d_{11}, d_{22} \in \mathbb{R}$.

We can always rewrite a linear combination of sine and cosine as a single sine with a phase shift as:

$$d_{22}\cos 2\psi + d_{11}\sin 2\psi = R\sin(2\psi + \delta),$$

where R and δ are constants determined by the coefficients d_{11} and d_{22}.

Expanding the right-hand side using the trigonometric identity we get

$$R\sin(2\psi + \delta) = R\sin 2\psi\cos\delta + R\cos 2\psi\sin\delta.$$

Comparing terms with the left-hand side $d_{22}\cos 2\psi + d_{11}\sin 2\psi$, we obtain

$$R\cos\delta = d_{11}, \qquad R\sin\delta = d_{22}.$$

From these relations:

$$R = \sqrt{d_{11}^2 + d_{22}^2}, \qquad \tan\delta = \frac{d_{22}}{d_{11}}.$$

Hence,

$$\boxed{\delta = \arctan\left(\frac{d_{22}}{d_{11}}\right)}.$$

The maxima and minima of $I^{2\omega}_{Y,PG3}$ occur when the sine term equals ± 1 or 0, respectively.

Maxima:

$$\sin^2(2\psi + \delta) = 1$$

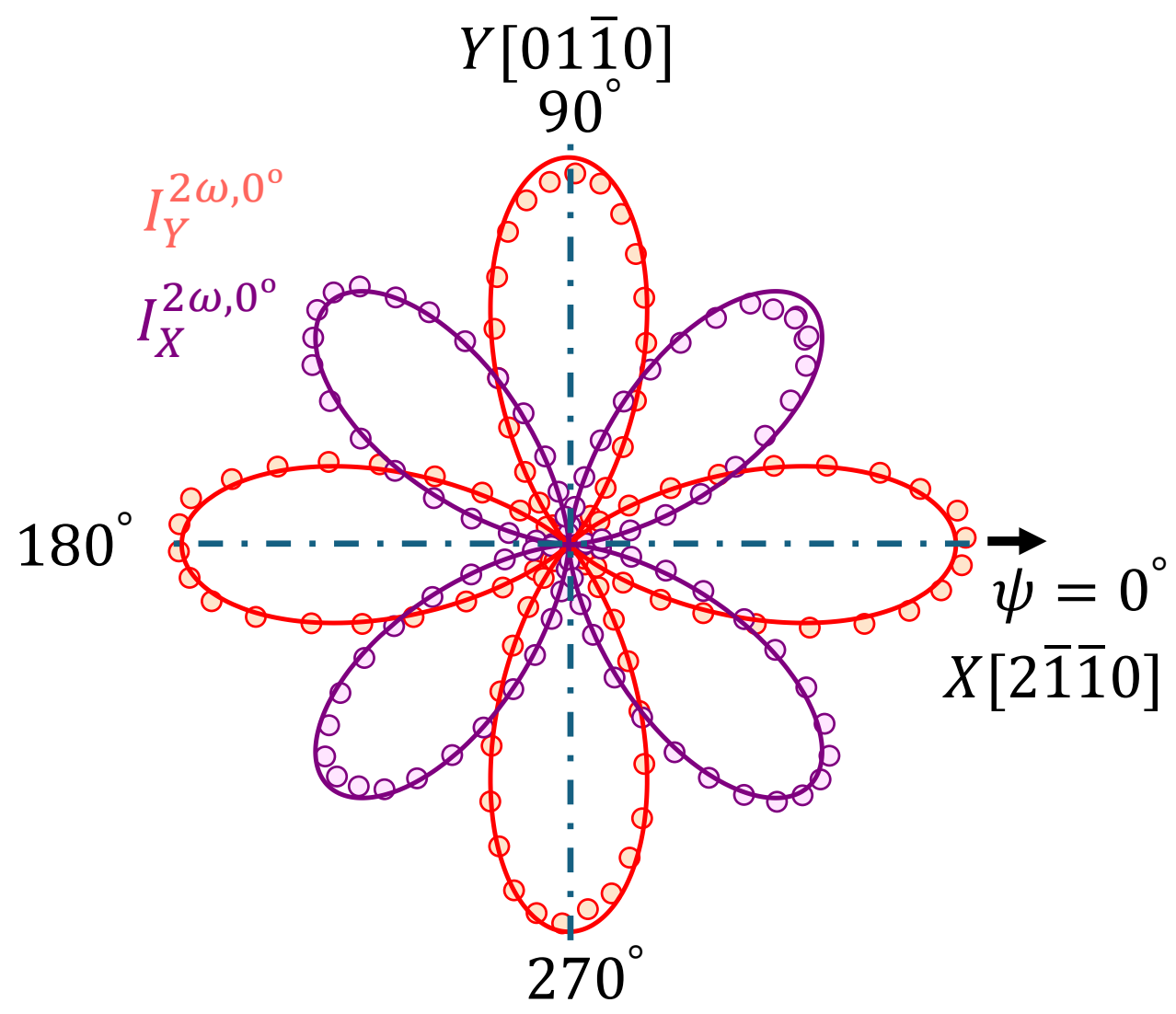

Figure S9: For the z-cut $LiNbO_3$ reference sample, the SHG intensity is plotted as a function of the incident polarization in the normal transmission geometry for analyzer $\parallel$ [2$\bar{1}\bar{1}$0] and analyzer $\perp$ [2$\bar{1}\bar{1}$0].

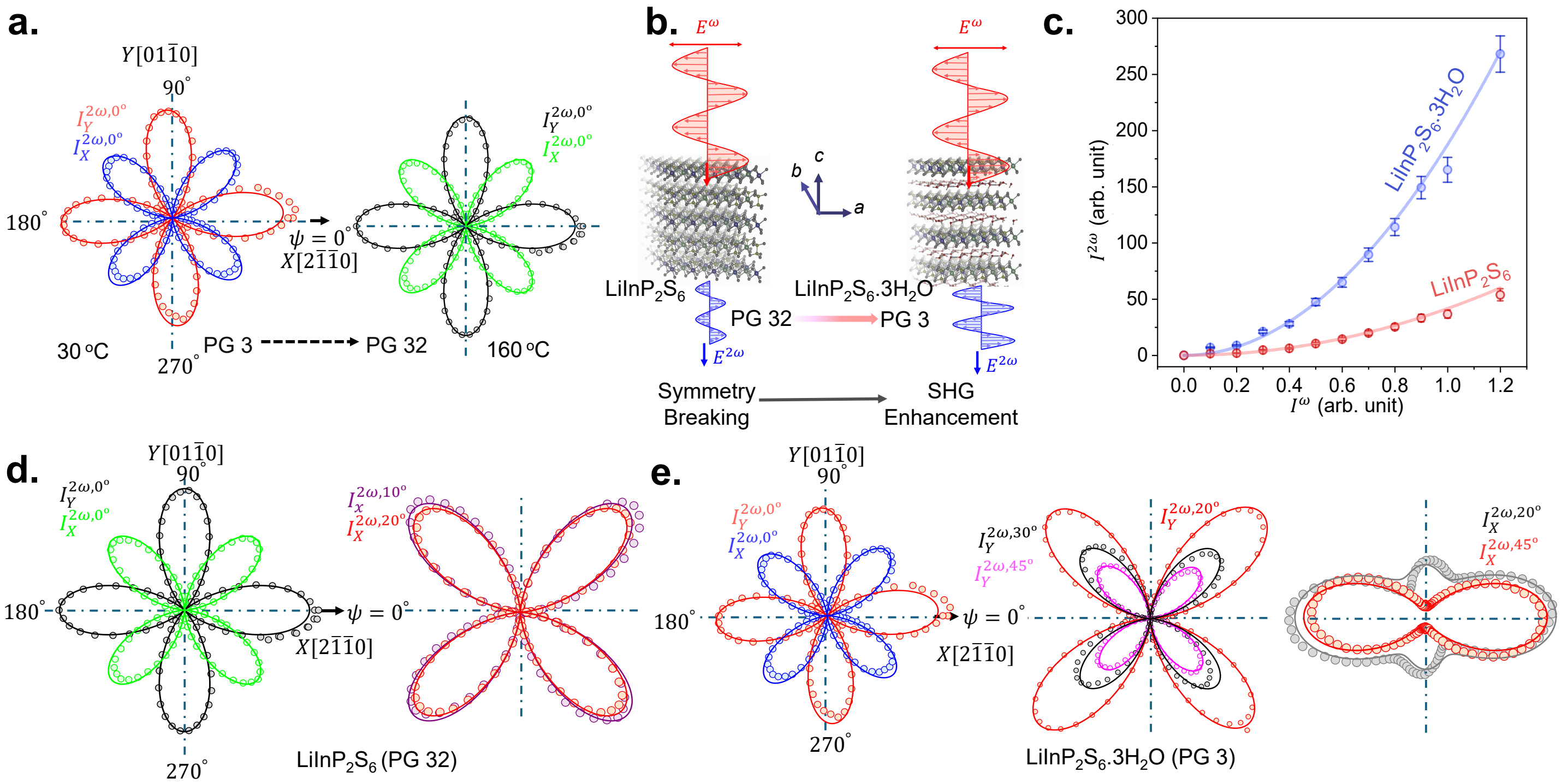

Figure S10: Nonlinear optical properties of $LiInP_2S_6$ and $LiInP_2S_6.3H_2O$: a. SHG polarimetries (circles) for normal incidence fitted (solid lines) to PG 3 (left) at RT and 160 °C, transition to higher symmetry PG 32 ∼160 °C. SHG polarimetry fits confirm symmetry lowering from PG 32 (anhydrous) to PG 3 (hydrated), consistent with DFT prediction. b. The reduction in symmetry leads to SHG enhancement for the hydrate phase compared to the $LiInP_2S_6$, as shown schematically. c. SHG power dependence (circles) for $LiInP_2S_6.3H_2O$ and $LiInP_2S_6$ single crystals in the normal transmission geometry, along with the expected quadratic fit (solid lines), confirming an order-of-magnitude enhancement in the hydrated phase compared to the $LiInP_2S_6$. Polarimetry plots of the SHG intensity as a function of the polarization of the incident light given by the polar angle, ψ, are shown in (d) $I_X^{2\omega,\alpha}(\psi)$ for s-polarized SHG and $I_Y^{2\omega,\alpha}(\psi)$ for p-polarized SHG for normal incidence and two incidence angles of the fundamental light, namely, $\alpha = 10°, 0°, 20°$ for $LiInP_2S_6$. The experimental data are shown as circles, while solid curves are theoretical fits based on point group 32, (e) SHG polarimetry for normal incidence and different oblique incidences, and fitting considering the point group 3.

$$\boxed{\psi_{\max} = \frac{\pi}{4} - \frac{\delta}{2} + \frac{k\pi}{2}}.$$

Minima:

$$\sin^2(2\psi + \delta) = 0,$$

$$\boxed{\psi_{\min} = -\frac{\delta}{2} + \frac{k\pi}{2}}$$

Here, $\delta/2$ represents the angular rotation of the four-lobed SHG polarimetry relative to the crystallographic axes, determined by the ratio of d_{22} and d_{11} as shown in the Figure S8 for $LiInP_2S_6$ and $LiInP_2S_6.3H_2O$. To extract the SHG tensor coefficients, systematic SHG polarimetry experiments were performed and analyzed using the open-source `#SHAARP` software package. The relative ratios of the nonlinear susceptibility tensor elements d_{ij} were obtained by simultaneously fitting the measured polar plots to the analytical model developed by Herman and Hayden, as described in the main text. The absolute values of the SHG coefficients were further determined by comparing the SHG intensities measured at normal incidence ($\alpha = 0°$) for $LiInP_2S_6$ and $LiInP_2S_6.3H_2O$ with those obtained from a reference z-cut $LiNbO_3$ crystal (see Figure S9) under identical experimental conditions.

Figure S10a shows representative SHG polarimetries (symbols) at room temperature and 160 °C, together with theoretical fits (solid lines) for PG 3 and PG 32. For SHG polarimetry in $LiInP_2S_6.3H_2O$ (left panel of Figure S10a), the best fit corresponds to the lower-symmetry PG 3, while above ∼160 °C-corresponding to $LiInP_2S_6$ (right panel of Figure S10a), the SHG polarimetry best fits with PG 32. The large SHG enhancement in PG 3 compared to PG 32 through symmetry lowering is shown as a schematic in Figure S10b).

The intensity of second-harmonic generation (SHG) is expected to scale quadratically with the incident optical fluence, following $I_{2\omega} = AI_{\omega}^2$. Experimentally, the fluence dependence of SHG from $LiInP_2S_6$ single crystals (thickness ∼63 µm) confirms this relation (Figure S10c), establishing the non-resonant, electric-dipole origin of the nonlinear response and large SHG enhancement in $LiInP_2S_6.3H_2O$.

Polarimetry at normal incidence shows excellent agreement with theoretical fits based on this tensor (see Figure S10d-e), confirming that the pristine crystal belongs to PG 32 symmetry, while the hydrated sample belongs to PG 3. Polarimetry at multiple oblique incidence angles (see Figure S10d-e) shows excellent correspondence between experiment and theoretical modeling using the Herman-Hayden formalism,[?] as implemented in the open-source #SHAARP software.[?, ?] The model fully accounts for multiple reflections of the ordinary and extraordinary SHG waves within the crystal.

7 Humidity-Dependent Stability and Reversible Switching of the SHG Response

To further evaluate the stability of the reversible SHG response, repeated heating-cooling cycles were performed on the same crystal while monitoring the SHG intensity at a fixed position. As shown in Figure S11, the SHG signal decreases during heating and recovers upon cooling, with the effect repeating over repeated cycles. We observed that, as hydration gradually returned, the SHG intensity developed noticeable spatial variation. To examine this effect, we performed spatially resolved SHG mapping over a $70\mu m \times 70\mu m$ area using a 20× objective on a WITec microscope. The measurements were carried out with a Ti:Sapphire Solstice Ace laser operating at a fundamental wavelength of 800 nm, and the SHG signal was collected at 400 nm at different stages of the experiment. The SHG signal was monitored at each stage, and the response was found to be reproducible over the measured cycles, with the intensity decreasing upon heating and recovering upon cooling. Although small cycle-to-cycle variations in intensity are observed, the overall response remains reversible, indicating that the SHG modulation is not a one-time thermal effect. These measurements support the stability of the water-intercalation-induced nonlinear optical response over multiple heating-cooling cycles. A controlled-humidity environment was constructed using a sealed incubation chamber equipped with a humidifier. The temperature and relative humidity

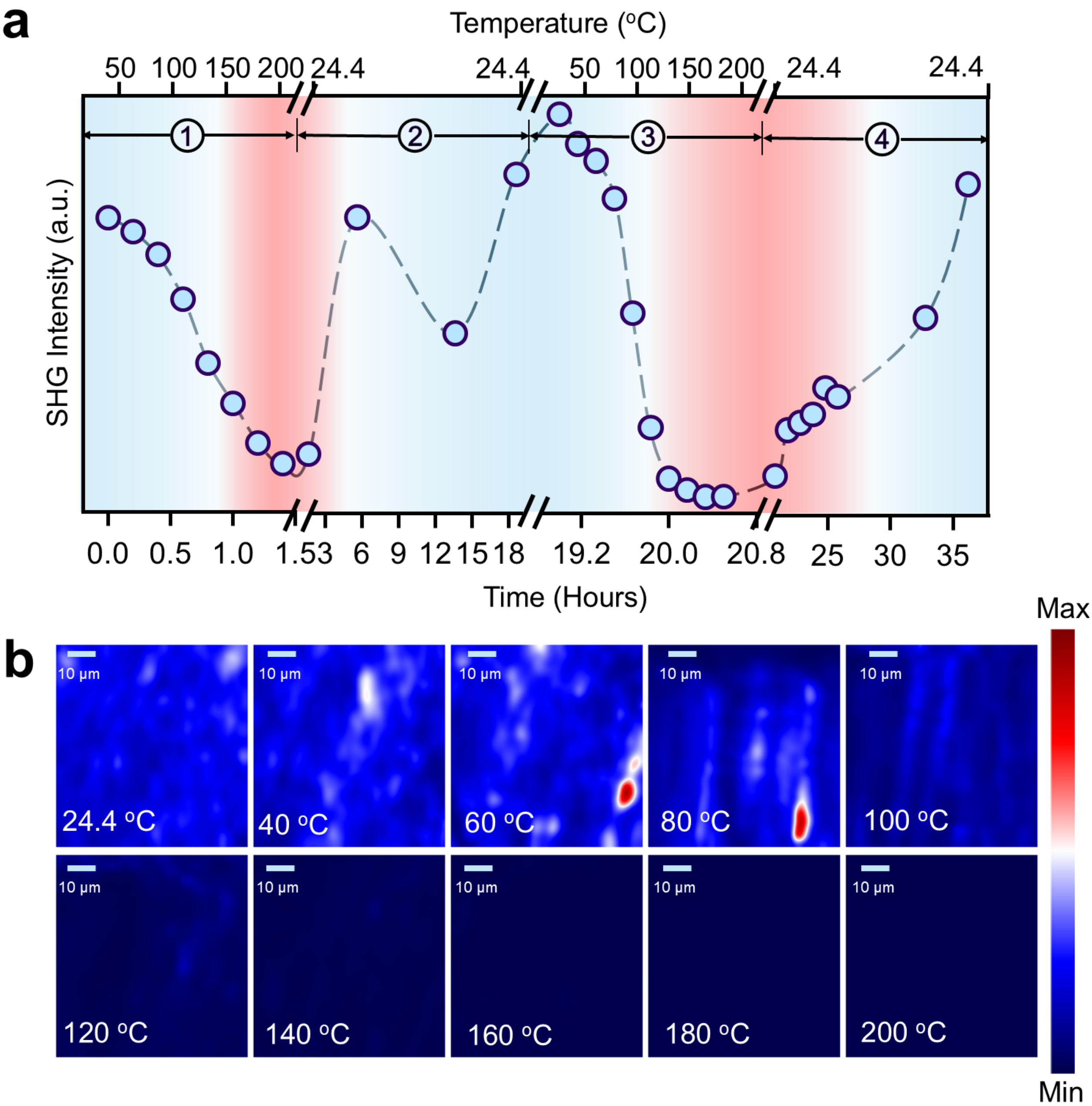

Figure S11: Temperature-dependent second-harmonic generation response during thermal cycling of the hydrated LIPS sample. (a) Integrated SHG intensity as a function of time during sequential heating and cooling cycles. The top axis indicates the applied temperature, while the breaks in the time axis mark discontinuities between separate measurement segments. The red-shaded regions indicate heating intervals, whereas the blue-shaded regions indicate cooling or low-temperature recovery intervals. The numbered regions denote different stages of the thermal response: ① the initial low-temperature hydrated state,② the first recovery stage after heating and cooling, ③ the second reheating cycle, and ④ the final cooling/recovery stage. The SHG intensity decreases strongly upon heating to an elevated temperature and partially recovers upon cooling, indicating a reversible but history-dependent change in the nonlinear optical response. (b) Spatial SHG intensity maps acquired at selected temperatures during the heating sequence. Each image shows the corresponding SHG map, plotted using a common minimum-to-maximum intensity scale to allow direct comparison across temperature. The SHG contrast is strongest at low temperature and progressively decreases with increasing temperature, becoming nearly suppressed above 120-140 °C. Scale bars are 10 μm.

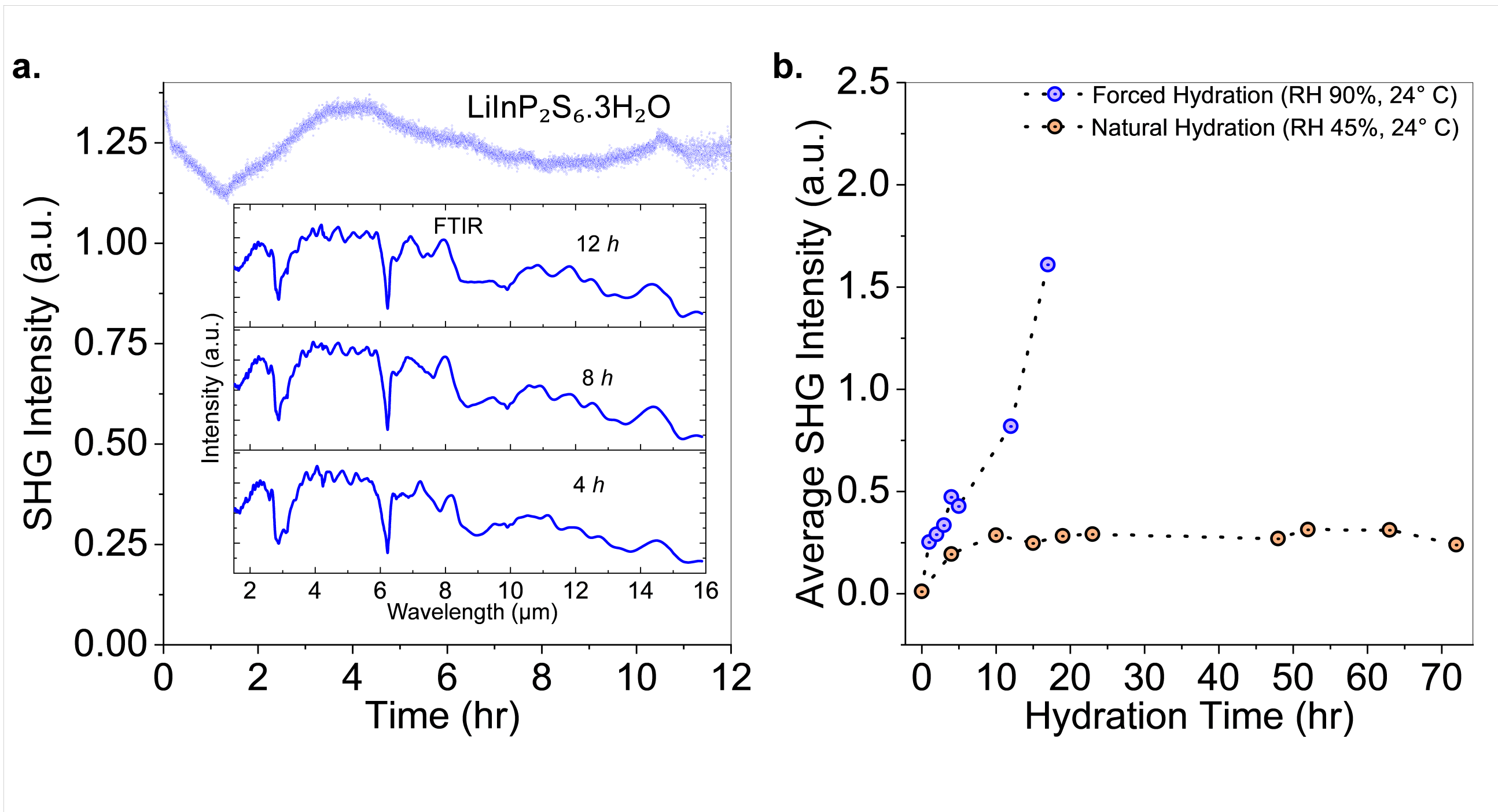

Figure S12: (a) Time-dependent SHG intensity of hydrated $LiInP_2S_6{\cdot}3H_2O$ measured under ambient conditions (∼45% relative humidity, room temperature) over 12 h, demonstrating the stability of the hydration-induced polar phase. The inset shows the corresponding FTIR spectra collected after 4, 8, and 12 h, indicating negligible change in the water-related vibrational modes. (b) Recovery of the SHG intensity of thermally annealed $LiInP_2S_6$ during forced hydration (∼90% relative humidity) and natural hydration under ambient conditions (∼45% relative humidity), showing substantially faster recovery kinetics under high-humidity conditions.

(RH) inside the chamber were continuously monitored using a calibrated humidity/temperature meter. During the hydration experiments, the samples were exposed to RH values in the range of 44–96% for durations of 1–5 days. The chamber was kept air-sealed during each exposure period, and the humidifier was operated intermittently to reach and maintain the target RH.

Care was taken to avoid water condensation inside the chamber. We found that operating the humidifier continuously for more than approximately 4-6 h after the atmosphere had reached saturation could lead to visible water droplet formation on the chamber walls and, in some cases, on the sample surface. Such direct condensation was observed to degrade the sample response. Therefore, after the desired RH was reached, the humidifier was turned off and the chamber was monitored closely to prevent precipitation or droplet formation during the exposure.

To establish the effect of humidity on the nonlinear optical response, SHG maps were acquired after controlled humidity exposures. The same 70 μm × 70 μm region was repeatedly measured throughout the experiment to minimize spatial variations between different measurements. Between humidity-control intervals, the sample was removed from the chamber and allowed to equilibrate for approximately 1 h under ambient laboratory conditions before SHG mapping. Control measurements showed that the SHG response remained relatively unchanged under ambient conditions at approximately 44% RH over the relevant measurement time scale. Therefore, changes in the SHG intensity observed after high-humidity exposure were attributed primarily to the controlled hydration environment rather than to ambient exposure during the mapping intervals. This procedure enabled the humidity-dependent SHG response to be tracked across multiple hydration and recovery cycles.

To investigate the environmental stability and controllability of the hydration-induced polar phase in $LiInP_2S_6{\cdot}3H_2O$, we performed additional time-dependent SHG, FTIR, and humidity-controlled rehydration measurements. Figure S12a shows the SHG intensity of the hydrated phase monitored continuously for

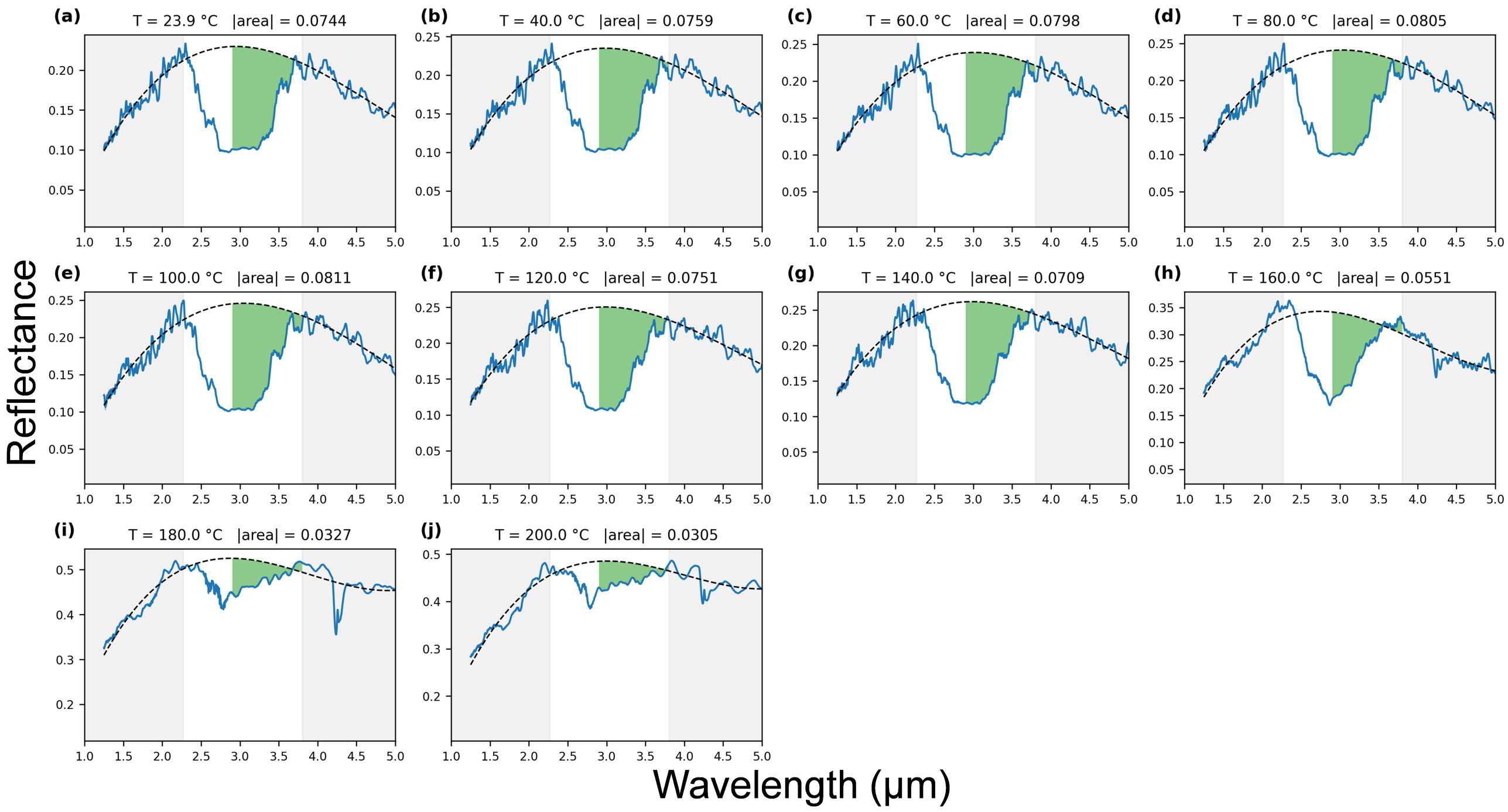

Figure S13: FTIR reflectance spectra measured from 23.9 °C to 200 °C are shown in panels (a)–(j). In each panel, the measured spectrum (solid blue line) and the fitted third-order polynomial background (black dashed line) are displayed. The integrated spectral area above the fitted background is highlighted in green, and the corresponding absolute peak area ($|A|$) is indicated in the panel title.

12 h under ambient laboratory conditions (∼45% relative humidity at room temperature). The SHG response remains nearly constant throughout the measurement period, indicating that the hydration-induced polar phase is stable under ambient conditions. Consistently, the corresponding FTIR spectra (inset of Figure S12a) collected after 4, 8, and 12 h show negligible changes in the water-related vibrational bands, confirming the stability of the intercalated water molecules within the lattice over the experimental timescale.

To further examine the reversibility of the hydration-driven nonlinear optical response, the hydrated sample was first thermally annealed at 200 °C to recover the low-SHG anhydrous phase. The annealed sample was subsequently exposed to different humidity environments while monitoring the SHG intensity as a function of time as shown in Figure S12b. Under forced hydration conditions (∼90% relative humidity at room temperature in a sealed humidified chamber), the SHG intensity progressively recovered and reached the same order of magnitude as the initial hydrated state within ∼20 h, demonstrating rapid rehydration-induced restoration of the polar phase. In contrast, under natural ambient hydration conditions (∼45% relative humidity), the SHG recovery process occurred over a substantially longer timescale, requiring several days to gradually approach the original hydrated-state intensity.

The pronounced dependence of the SHG recovery kinetics on environmental humidity demonstrates that the nonlinear optical response of $LiInP_2S_6$ can be reversibly and controllably modulated through water intercalation. These results further support that the observed switching behavior originates from reversible hydration within the van der Waals gaps.

8 Hydration-Correlated SHG Response

To clarify the role of water content in the SHG response, complementary quantitative FT-IR reflectance measurements were performed on the same samples. Following Clark et al. [?], the broad H_2O combination band centered near 3 μm was used as a proxy for absorbed water. After Savitzky–Golay smoothing, the spectral background was fit with a third-order polynomial (black dashed line) using only the regions on either side of the band and extrapolated across it as shown in Figure S13. The integrated area between the

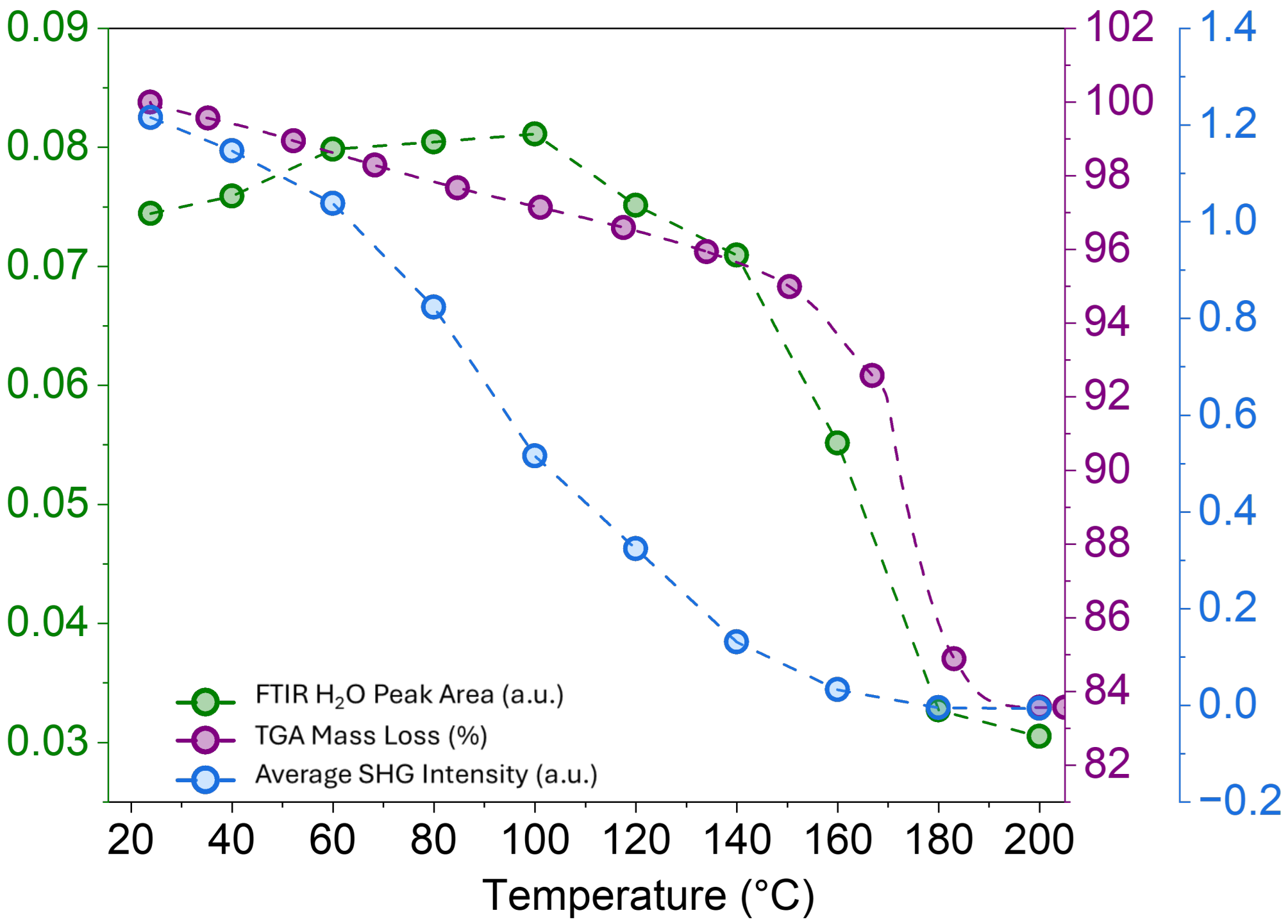

Figure S14: Temperature dependence of the SHG intensity, integrated area of the water-absorption band extracted from FTIR reflectance spectra, and weight loss measured by TGA for hydrated $LiInP_2S_6$ during heating from 24 °C to 200 °C. The gradual dehydration of $LiInP_2S_6$ upon heating is accompanied by concurrent changes in all three quantities, revealing a correlation between water content, SHG response, and mass loss.

spectrum and its smoothen background, evaluated from 2.9 to 3.8 μm and shaded green region, was taken as a measure of the water content of $LiInP_2S_6$. The procedure was repeated at every temperature from 24 °C to 200 °C. We compared the temperature-dependent dehydration behavior using FTIR reflectance, TGA, and SHG microscopy. TGA measures mass loss directly, while the integrated water-related FTIR feature tracks the loss of vibrational signatures associated with water. The close agreement between these two measurements confirms that the FTIR spectral change is linked to water removal and the hydrated-to-anhydrated phase transition (Figure S14). The SHG intensity also decreases upon heating, but the raw SHG signal drops more sharply than the TGA and FTIR trends. This suggests that the SHG response is not governed solely by the amount of water entering or leaving the structure, but also by hydration-induced changes in the local bonding environment that lead to noncentrosymmetry.

9 Computational Investigation of $LiInP_2S_6 \cdot 3\,H_2O$ via Separated $LiInP_2S_6$ Layers and Interlayer Water

To better understand the interaction between $LiInP_2S_6$ layers and the interlayer water in $LiInP_2S_6 \cdot 3\,H_2O$, we performed calculations on their separated structures without permitting any atomic relaxation. The separated structures are shown in Figure S15a-c, where the structures have been translated in the unit cell for clarity. The polar arrangement of the separated interlayer water structure leads to an obvious electric field (electrostatic potential gradient) in the vacuum regions of the simulation cell (Figure S15d). The c-axis unit cell average polarization density is 1.0 $\mu C/cm^2$ Using a dipole-layer correction that applies a compensating smooth step function, we find that a large applied electric field of 9.8 MV/mm is required to counter the unit cell average field of the water molecules. This can be seen as the 1.92 eV shift in the work function over the length of the unit cell in Figure S15c. The calculated static SHG tensors for the separated $LiInP_2S_6$ layers, the interlayer water, and bulk $LiInP_2S_6 \cdot 3\,H_2O$ are presented together for

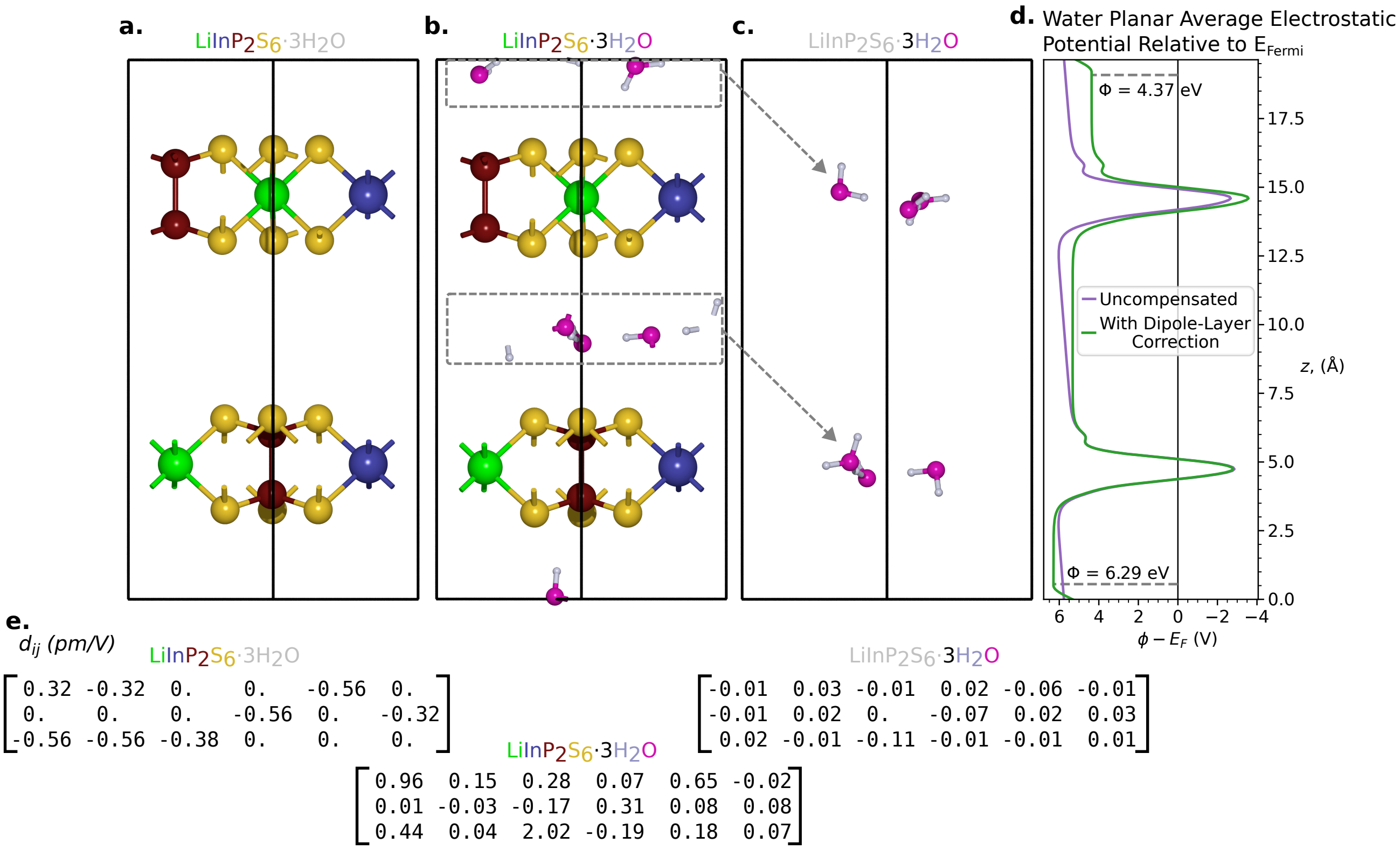

Figure S15: (a) Crystal structure of $LiInP_2S_6$. (b) Crystal structure of hydrated $LiInP_2S_6 \cdot 3\,H_2O$. (c) Interlayer water molecules extracted from the hydrated structure and shifted away from the periodic boundaries for clarity. (d) Electrostatic potential of the interlayer water calculated without and with the dipole-layer correction. (e) Comparison of the SHG tensors for $LiInP_2S_6 \cdot 3\,H_2O$ and the separated $LiInP_2S_6$ and water layers.

comparison in Figure S15e.

10 Interlayer Migration of Lithium

To examine the interlayer migration of lithium, we performed simulated annealing calculations on a $2 \times 2 \times 1$ supercell of the $LiInP_2S_6 \cdot 3\,H_2O$ structure, in which one lithium atom was displaced into the interlayer region. Only 8× trial runs were performed to reduce the computational cost associated with the DFT-level optimization of the final 152-atom cells. The lowest-energy structure was taken as the endpoint of the lithium migration pathway. The starting-point structure was generated by placing the lithium atom back into its sulfur-coordinated site within the $LiInP_2S_6$ layer and subsequently re-relaxing the structure. The migration pathway was sampled using 16 interior images in a nudged elastic band (NEB) calculation initialized by linear interpolation between the starting and ending structures. Unfortunately, the NEB calculation exhibited poor convergence owing to the highly tortuous reaction pathway, which involved multiple water-molecule rotations after the lithium atom left the $LiInP_2S_6$ layer but before reaching the equilibrium endpoint. Although the NEB optimization was terminated after 305 iterations (4880 image calculations), the resulting reaction pathway still provides an upper-bound estimate of the migration energy profile. The equilibrium starting structure, highest-energy image, and equilibrium ending structure, together with the insufficiently converged NEB reaction pathway, are shown in Figure S16a,b. These results indicate that the lithium migration barrier is associated with a transition state (TS) in which the lithium atom is nearly coplanar with the three outermost coordinating sulfur atoms. Since the precise reaction pathway is of less interest than the migration barrier relative to the starting and ending energies, a dimer-method search for the TS was initialized using the highest-energy NEB image. A simplified lithium migration pathway, including only the relative energies of the intralayer lithium starting point, the TS, and the interlayer water-coordinated endpoint, along with the TS structure, is shown in Figure S16d,e.

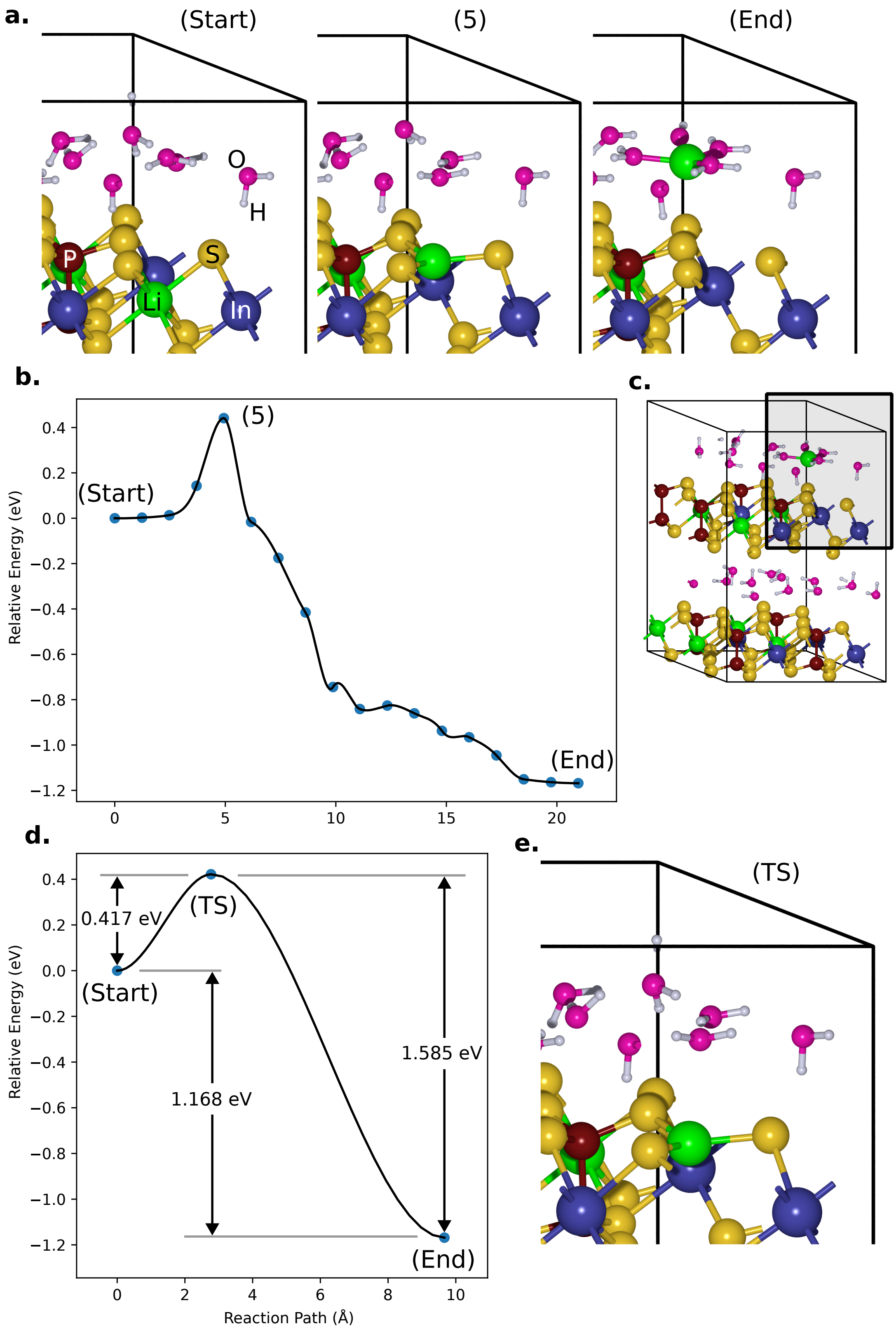

Figure S16: (a) Zoomed in views of the lithium atom in the relaxed starting and ending configurations with highest energy structure (image 5) along the MEP found via NEB. (b) Energy along the reaction path (c) Overall view of the 2x2x1 supercell used for calculations of the intra- to interlayer lithium migration. (d) A simplified reaction path of the starting, transition state (TS), and ending energy view of lithium migration. (e) The transition state structure found via dimer search. Energy interpolation in (b) and (d) between images uses cubic polynomials fit to energies and energy derivatives from force-tangent projections.

11 $LiInP_2S_6 \cdot 3\,H_2O$ Atomic Coordinates

The $LiInP_2S_6 \cdot 3\,H_2O$ atomic coordinates are tabulated in S4

Table S2: Vibrational modes were calculated for the lowest energy $LiInP_2S_6.3H_2O$ structure where the $LiInP_2S_6$ layers were constrained to PG 3. The low-frequency imaginary modes are artifacts of the constrained relaxation.

Mode	meV	cm^{-1}	λ (μm)	Intensity ((D/Å)^2 amu^{-1})		Mode	meV	cm^{-1}	λ (μm)	Intensity ((D/Å)^2 amu^{-1})
0	3.9i	31.1i	321.5i	7.37		57	33.7	271.8	36.8	746.36
1	2.6i	21.1i	473.9i	641.12		58	33.8	272.5	36.7	54.02
2	1.2i	10.0i	1000.0i	2204.01		59	33.9	273.5	36.6	53.36
3	0.9i	7.1i	1408.5i	269.34		60	33.9	273.6	36.6	593.98
4	0.9	7.3	1369.9	1505.68		61	35.5	286.2	34.9	208.46
5	1.5	12.4	806.5	364.34		62	39.4	317.7	31.5	9.37
6	2.0	16.0	625.0	799.84		63	40.0	322.9	31.0	448.30
7	3.3	26.7	374.5	102.61		64	41.2	332.2	30.1	56.29
8	3.6	28.7	348.4	488.53		65	41.5	334.9	29.9	966.32
9	4.1	33.4	299.4	469.17		66	42.9	345.7	28.9	10.45
10	7.8	63.2	158.2	116.40		67	43.8	353.0	28.3	26.54
11	8.1	65.0	153.8	507.36		68	44.6	359.6	27.8	1117.19
12	8.7	70.3	142.3	311.03		69	44.9	361.9	27.6	635.00
13	9.2	73.9	135.3	238.09		70	45.4	366.5	27.3	1900.28
14	10.5	84.9	117.8	28.10		71	49.9	402.2	24.9	22.71
15	10.8	87.3	114.5	86.94		72	54.4	438.5	22.8	22.33
16	11.2	90.4	110.6	133.32		73	54.7	441.3	22.7	44.59
17	11.7	94.0	106.4	147.47		74	56.7	457.4	21.9	0.51
18	12.2	98.1	101.9	162.41		75	57.4	463.2	21.6	1.13
19	12.3	99.5	100.5	118.44		76	62.9	507.3	19.7	23.84
20	12.8	103.4	96.7	113.84		77	66.4	535.6	18.7	228.81
21	13.9	112.1	89.2	49.93		78	67.0	540.4	18.5	456.16
22	15.3	123.5	81.0	121.52		79	67.2	541.6	18.5	133.09
23	15.8	127.1	78.7	8.14		80	67.7	545.9	18.3	1222.50
24	16.1	130.3	76.7	47.94		81	67.7	546.0	18.3	27.98
25	16.2	130.8	76.5	114.58		82	68.0	548.3	18.2	311.78
26	16.3	131.7	75.9	519.47		83	68.0	548.5	18.2	1018.33
27	16.4	132.6	75.4	418.58		84	68.1	549.6	18.2	182.20
28	19.1	154.1	64.9	1636.80		85	68.2	550.3	18.2	116.72
29	19.3	155.3	64.4	532.06		86	68.4	551.9	18.1	329.34
30	19.7	158.9	62.9	3075.38		87	68.6	553.6	18.1	289.88
31	19.7	159.1	62.8	2138.60		88	73.3	590.9	16.9	7.16
32	20.3	163.9	61.0	162.30		89	77.0	621.0	16.1	55.22
33	20.4	164.7	60.7	540.21		90	93.0	750.0	13.3	5.69
34	20.9	168.5	59.3	143.11		91	96.2	775.5	12.9	172.85
35	21.1	170.5	58.6	52.31		92	107.0	862.9	11.6	48.72
36	22.0	177.8	56.2	188.20		93	109.6	883.7	11.3	10.87
37	23.5	189.3	52.8	46.62		94	123.0	992.2	10.1	70.15
38	25.0	201.9	49.5	28.61		95	130.1	1049.3	9.53	2.55
39	25.2	202.9	49.3	65.37		96	194.2	1566.2	6.38	0.35
40	25.4	204.9	48.8	293.66		97	197.0	1589.0	6.29	26.01
41	25.6	206.4	48.5	93.45		98	199.3	1607.6	6.22	2.85
42	28.4	228.9	43.7	475.91		99	199.5	1609.4	6.21	157.26
43	28.5	229.9	43.5	111.89		100	205.0	1653.5	6.05	6.19
44	29.8	240.4	41.6	1444.29		101	205.1	1654.6	6.04	39.26
45	29.9	240.8	41.5	45.57		102	375.2	3026.6	3.30	14.09
46	30.5	245.7	40.7	122.79		103	376.5	3036.5	3.29	186.61
47	30.6	246.9	40.5	286.36		104	393.8	3175.9	3.15	75.58
48	30.7	247.9	40.3	56.16		105	394.4	3180.8	3.14	1.79
49	30.9	248.9	40.2	53.84		106	399.4	3221.1	3.10	9.06
50	30.9	249.1	40.1	649.10		107	403.1	3251.5	3.07	2.97
51	31.4	253.4	39.5	1069.85		108	456.4	3681.0	2.72	34.42
52	31.7	255.8	39.1	278.99		109	457.1	3687.1	2.71	3.18
53	32.2	259.5	38.5	457.15		110	460.7	3715.6	2.69	7.44
54	32.2	259.8	38.5	331.52		111	461.7	3723.5	2.69	0.40
55	32.4	261.3	38.3	98.33		112	465.5	3754.7	2.66	74.28
56	32.5	261.8	38.2	333.81		113	466.3	3760.6	2.66	3.88

Table S3: List of amplitude, broadening parameter, and center energy for the Lorentz oscillator utilized to quantify the dielectric function.

Type	Amplitude, A	Broadening parameter, γ (eV)	Energy, E_0 (eV)
$\epsilon_{\text{ordinary}}$	2.31	0.56	4.01
$\epsilon_{\text{ordinary}}$	0.20	0.22	2.93
$\epsilon_{\text{ordinary}}$	943.00	0.07	0.06
$\epsilon_{\text{ordinary}}$	2.93	0.07	2.03
$\epsilon_{\text{ordinary}}$	4.34	1.81	4.91
$\epsilon_{\text{ordinary}}$	3.27	1.91	6.39
$\epsilon_{\text{extraordinary}}$	2.36	0.73	4.14
$\epsilon_{\text{extraordinary}}$	1.31	0.67	2.63
$\epsilon_{\text{extraordinary}}$	1000.00	0.08	0.10
$\epsilon_{\text{extraordinary}}$	50.64	0.26	1.53
$\epsilon_{\text{extraordinary}}$	1.34	0.85	5.04
$\epsilon_{\text{extraordinary}}$	3.59	0.60	5.88

Lattice Parameter	a (Å)	b (Å)	c (Å)	α (°) x	β (°)	γ (°)
	6.103818	6.103818	19.701264	90	90	120
Atom	X_a	X_b	X_c	R_x (Å)	R_y (Å)	R_z (Å)
H1	0.675891	0.284540	0.252818	3.257127	1.504097	4.980829
H2	0.372640	0.393594	0.240304	1.073316	2.080560	4.734292
H3	0.310258	0.141515	0.203246	1.461868	0.748059	4.004202
H4	0.384183	0.783986	0.257894	-0.047669	4.144197	5.080841
H5	0.125230	0.529833	0.243375	-0.852623	2.800728	4.794795
H6	0.606632	0.392031	0.748673	2.506329	2.072302	14.749798
H7	0.933273	0.437049	0.807096	4.362694	2.310266	15.900810
H8	0.174967	0.155797	0.734166	0.592489	0.823555	14.464007
H9	0.893599	0.241233	0.750130	4.718146	1.275172	14.778505
H10	0.956490	0.877422	0.751881	3.160429	4.638105	14.813002
H11	0.498197	0.530461	0.705049	1.421987	2.804048	13.890353
H12	0.826655	0.218906	0.305493	4.377673	1.157152	6.018596
Li1	0.333333	0.666667	0.499649	0.000000	3.524041	9.843708
Li2	0.000000	0.000000	0.999802	0.000000	0.000000	19.697359
O1	0.838671	0.283893	0.259623	4.252682	1.500676	5.114903
O2	0.385899	0.237220	0.244601	1.631483	1.253959	4.818946
O3	0.457478	0.418027	0.743898	1.516579	2.209718	14.655740
O4	0.307299	0.633923	0.229377	-0.058980	3.350954	4.519023
O5	0.990379	0.030007	0.727633	5.953516	0.158616	14.335294
O6	0.874070	0.390804	0.760505	4.142467	2.065814	14.982901
P1	0.333333	0.666667	0.057264	0.000000	3.524041	1.128181
P2	0.333333	0.666667	0.943881	0.000000	3.524041	18.595658
P3	0.000000	0.000000	0.443748	0.000000	0.000000	8.742389
P4	0.000000	0.000000	0.557127	0.000000	0.000000	10.976107
S1	0.011680	0.340867	0.083897	-0.969000	1.801842	1.652871
S2	0.320040	0.324030	0.415073	0.964555	1.712843	8.177466
S3	0.675970	0.996010	0.415073	1.086266	5.264970	8.177466
S4	0.003990	0.679960	0.415073	-2.050822	3.594311	8.177466
S5	0.321945	0.996112	0.583632	-1.074950	5.265511	11.498279
S6	0.003888	0.325833	0.583632	-0.970682	1.722372	11.498279
S7	0.674167	0.678055	0.583632	2.045632	3.584240	11.498279
S8	0.659133	0.670814	0.083897	1.975968	3.545963	1.652871
S9	0.329704	0.342754	0.915282	0.966403	1.811817	18.032220
S10	0.013049	0.670296	0.915282	-1.966031	3.543224	18.032220
S11	0.657246	0.986951	0.915282	0.999628	5.217083	18.032220
S12	0.329186	0.988320	0.083897	-1.006969	5.224319	1.652871
In1	0.666667	0.333333	0.000000	3.051909	1.762021	0.000000
In2	0.666667	0.333333	0.499607	3.051909	1.762021	9.842895

Table S4: The lattice parameters, fractional atomic coordinates (X), and Cartesian atomic coordinates (R) for the $LiInP_2S_6 \cdot 3\,H_2O$ structure.
